# Functional, Structural and Biochemical Features of Plant Serinyl-Glutathione Transferases

**DOI:** 10.3389/fpls.2019.00608

**Published:** 2019-05-22

**Authors:** Elodie Sylvestre-Gonon, Simon R. Law, Mathieu Schwartz, Kevin Robe, Olivier Keech, Claude Didierjean, Christian Dubos, Nicolas Rouhier, Arnaud Hecker

**Affiliations:** ^1^Interactions Arbres-Microorganismes, Institut National de la Recherche Agronomique, Université de Lorraine, Nancy, France; ^2^Department of Plant Physiology, Umeå Plant Science Centre, Umeå University, Umeå, Sweden; ^3^Centre National de la Recherche Scientifique, Cristallographie, Résonance Magnétique et Modélisations, Université de Lorraine, Nancy, France; ^4^Biochimie et Physiologie Moléculaire des Plantes (BPMP), INRA, CNRS, SupAgro-M, Université de Montpellier, Montpellier, France

**Keywords:** photosynthetic organisms, phylogeny, structure, glutathione transferases, ligandin property, secondary metabolism, xenobiotic detoxification

## Abstract

Glutathione transferases (GSTs) belong to a ubiquitous multigenic family of enzymes involved in diverse biological processes including xenobiotic detoxification and secondary metabolism. A canonical GST is formed by two domains, the N-terminal one adopting a thioredoxin (TRX) fold and the C-terminal one an all-helical structure. The most recent genomic and phylogenetic analysis based on this domain organization allowed the classification of the GST family into 14 classes in terrestrial plants. These GSTs are further distinguished based on the presence of the ancestral cysteine (Cys-GSTs) present in TRX family proteins or on its substitution by a serine (Ser-GSTs). Cys-GSTs catalyze the reduction of dehydroascorbate and deglutathionylation reactions whereas Ser-GSTs catalyze glutathione conjugation reactions and eventually have peroxidase activity, both activities being important for stress tolerance or herbicide detoxification. Through non-catalytic, so-called ligandin properties, numerous plant GSTs also participate in the binding and transport of small heterocyclic ligands such as flavonoids including anthocyanins, and polyphenols. So far, this function has likely been underestimated compared to the other documented roles of GSTs. In this review, we compiled data concerning the known enzymatic and structural properties as well as the biochemical and physiological functions associated to plant GSTs having a conserved serine in their active site.

## Introduction

Glutathione transferases (GSTs), formerly glutathione S-transferases, constitute a ubiquitous multigenic superfamily of enzymes that conjugate the tripeptide glutathione (γ-Glu-Cys-Gly) on a broad range of molecules. They catalyze the nucleophilic attack of reduced glutathione (GSH) on the electrophilic centers of these molecules. The omnipresence of these enzymes in all types of organisms highlights an ancient origin as well as fundamental functions preserved during evolution. GSTs were discovered in the early 1960s through their GSH-conjugating activity in cellular extracts from rat liver incubated with sulfobromophthalein, chloronitrobenzenes or halogenated aromatic molecules (Booth et al., 1961; Combes and Stakelum, 1961). Later on, this conjugating activity was identified from plant extracts (sorghum and corn) using herbicides like atrazine or triazine derivatives (Frear and Swanson, 1970; Lamoureux et al., 1970). The interest for these GSH-conjugation reactions in plants was high in the 1980s, particularly concerning the enzymatic properties of cereal GSTs in connection with the detoxification of herbicides (Shah et al., 1986; Wiegand et al., 1986). Accordingly, GSTs are generally strongly induced in response to biotic and abiotic stresses, which coincides with cellular roles in primary and secondary metabolisms, in stress tolerance or cell signaling, and in xenobiotic detoxification by acting as phase II enzymes (Jakoby, 1978; Wiegand et al., 1986; Gonzalez et al., 2018). During the detoxification process, GSTs, which represent the most important classes of conjugating enzymes, conjugate phase I-activated molecules (or toxic molecules that are already activated) with GSH. Conjugation reactions are only performed by specific GST members, i.e., those having usually a conserved serine or a tyrosine (in mammals) in their active site. Those having notably a cysteine residue lack this property. The conjugation step has several benefits in the detoxification process, including a decrease of the reactivity and toxicity of the molecules, as well as an increase of their solubility. Likewise, the addition of large anionic groups such as GSH detoxifies reactive electrophiles and produces polar molecules that cannot diffuse across membranes. These molecules are then recognized and actively transported by ATP-binding cassette transporters (ABC-transporters), also known as phase III proteins (Keppler, 1999). ABC transporters carry out the ATP-dependent transport of a large variety of hydrophobic molecules and thus participate in exocytosis (animals) or sequestration in the vacuole and/or in the cell wall (plants and fungi) of phase II products (Coleman et al., 1997).

Even though most of the studies published over the past years focused on GSTs catalyzing the addition of glutathione, other catalytic activities have been described. For instance, numerous GSTs act as GSH-dependent peroxidases by reducing organic hydroperoxides (Tang and Tu, 1994; Marrs, 1996; Hurst et al., 1998) whereas others perform isomerisation reactions. The zeta GSTs catalyze the *cis-trans* isomerisation of maleylacetoacetate into fumarylacetoacetate, as part of the tyrosine degradation pathway (Thom et al., 2001; Fernandez-Canon et al., 2002). In addition to their involvement in GSH-conjugation, several GSTs also catalyze the opposite reaction, i.e., the removal of glutathione from small molecules. This reaction will be referred to as deglutathionylation. Note that this term is also used for the reduction of glutathione adducts on protein cysteine residues, as catalyzed by another family of GSH-dependent proteins called glutaredoxins (GRXs) (Rouhier et al., 2008). Although human GSTO1-1 was shown to catalyze the deglutathionylation of peptides/proteins such as glutathionylated β-actin (Menon and Board, 2013), this has been rarely observed among GSTs. The capacity of catalyzing deglutathionylation reactions is linked to the existence of a catalytic cysteinyl residue instead of the active site serine or tyrosine residues. This residue is notably present in the bacterial-specific Beta GSTs (GSTBs); in Omega GSTs (GSTOs) found in mammals, insects, and fungi (Board et al., 2000; Kim et al., 2006; Yamamoto et al., 2009; Meux et al., 2013); in the plant-specific Lambda GSTs (GSTLs); and in glutathionyl-hydroquinone reductases (GHRs), also known as Xi GSTs (GSTX), which are found in bacteria, fungi, archaea, and plants (Xun et al., 2010; Meux et al., 2011; Lallement et al., 2015; Schwartz et al., 2016). However, with a few exceptions, the physiological role of these enzymes is poorly documented. In addition to being involved in deglutathionylation, as mentioned above, human GSTOs may be involved in arsenic biotransformation, reducing methyl and dimethyl arsenate forms (Zakharyan et al., 2001; Burmeister et al., 2008). Plant GSTLs may be involved in the metabolism or trafficking of flavonoids (Dixon and Edwards, 2010b). GHRs are involved in the catabolism of chlorinated quinones and in lignin degradation through the deglutathionylation of metabolic intermediates (Reddy and Gold, 2001; Masai et al., 2003; Huang et al., 2008; Meux et al., 2011).

In addition to these catalytic properties, some GSTs possess the property to bind ligands also referred to as ligandin properties. It consists of the binding of small hydrophobic molecules either at the catalytic site or in a specific ligandin site (L-site) for their transport or storage. This non-catalytic property has been documented in plants for the transport/binding of hydrophobic xenobiotic molecules, of endogenous compounds such as oxylipins and flavonoids (anthocyanins, proanthocyanidins) as well as of phytohormones such as auxin and cytokinin (Zettl et al., 1994; Gonneau et al., 1998), suggesting a possible role of GSTs in cell signaling and/or in plant growth and development (Smith et al., 2003; Kitamura et al., 2004; Gong et al., 2005; Moons, 2005; Ahmad et al., 2017).

Overall, although the biochemical (catalytic and ligandin) properties of representative members from almost all GST classes have been studied, sometimes extensively, the physiological role of most of them remains to be identified, essentially because the existence of several close isoforms in given classes may have hampered their characterization by reverse-genetic approaches. Therefore, having focused recently on the biochemical and structural properties of Cys-GSTs (Lallement et al., 2014), the objective of this review is to inventory the known properties and functions of Ser-GSTs in photosynthetic organisms.

### History and Classification of the GSTs

Over the past years, the GST classification has constantly evolved concomitantly to the increase of the genomic resources available, and to the identification and characterization of new isoforms and classes. First discovered in rat, GSTs were characterized initially in mammals and subsequently in insects, plants, fungi, and bacteria. In mammals, GSTs were originally classified as cytosolic, mitochondrial and membrane-associated GSTs, the latter being subdivided into microsomal GSTs and leukotriene C_4_ synthetases (Kraus, 1980; Hayes and Pulford, 1995). The same three subfamilies were renamed later as soluble GSTs, kappa GSTs and membrane-associated proteins in eicosanoids and glutathione metabolism (MAPEG), respectively (Jakobsson et al., 1999). However, on the basis of their immunological cross-reactivity and sequence relatedness, mammalian GSTs were also classified into the alpha, mu, pi, sigma, theta, and zeta classes (Mannervik et al., 1985; Dixon et al., 1998; Hayes and McLellan, 1999). At the time, most non-mammalian GSTs were placed in the heterogeneous theta class (Buetler and Eaton, 1992). For plant GSTs, the first classification introduced was based on sequence analogy and on the intron-exon structure of the genes. Subsequently, three and then four distinct types of plant GSTs were recognized including type I (GSTs with herbicide-detoxifying activity), type II (GSTs close to the mammalian zeta GSTs), type III (consisted mainly of auxin-induced GSTs), and type IV (GSTs similar to classical mammalian theta enzymes) isoforms (Droog et al., 1995; Droog, 1997). In fact, with the accumulation of biochemically characterized plant GSTs in the late 1990s, it appeared that some plant GSTs clearly grouped with specific mammalian GSTs, whereas others seemed plant-specific. Together with the release of the genome of *Arabidopsis thaliana*, this contributed to the establishment of a refined phylogenetic classification in plants using the principle of Greek-letter designations, which was widely used for non-plant GSTs (Dixon et al., 1998). GSTs are designated by using a 2 letter-code corresponding to the species (At for *A. thaliana*) followed by the 3 letters “GST,” a Greek or Latin letter designating the class, and a number distinguishing members of the same class. Thus, in *A. thaliana*, isoform 1 of the Phi (F) class is designated by “AtGSTF1”. This classification introduced in plants the Phi (replacing former Type I), Zeta (replacing former Type II), Tau (replacing former Type III), and Theta (replacing former Type IV) classes as well as two groups more distantly related to other known plant GSTs forming the Lambda (L) and dehydroascorbate reductase (DHAR) classes (Dixon et al., 2002). The last phylogenetic study performed a few years ago using well-annotated genomes of terrestrial plants (*A. thaliana, Hordeum vulgare, Oryza sativa, Physcomitrella patens, Pinus tabulaeformis, Populus trichocarpa*, and *Solanum lycopersicum*) and selecting only proteins possessing the two regular N- and C-terminal domains (see below), led to the identification of 14 GST classes: phi (F), tau (U), theta (T), zeta (Z), lambda (L), hemerythrin (H), iota (I), ure2p, glutathionyl-hydroquinone reductase (GHR), elongation factor 1B gamma (EF1Bγ), DHAR, tetrachlorohydroquinone dehalogenase (TCHQD), metaxin, and microsomal prostaglandin E synthase type-2 (mPGES-2) (Lallement et al., 2014). Some of these classes are found among different kingdoms, such as Zeta or Theta classes whereas Lambda, Tau and DHAR classes are specific to plants. The Phi class is sometimes presented in the literature as specific to the plant kingdom but similar sequences have been identified in some fungi, bacteria, and protists (Morel et al., 2013; Munyampundu et al., 2016). Although it has some limitations, the primary sequence remains to date the most convenient criterion for classifying these proteins.

The evolutionary history of GSTs seems relatively complex and several scenarios have been proposed. Because Theta class GSTs were present in bacteria, the first model of evolution, dating from the early 1990s, proposed that canonical (soluble) GSTs of plants, animals, and fungi have evolved from this ancestral gene as a result of duplications followed by divergent evolution (Pemble and Taylor, 1992). In subsequent years, this model was discarded by taking into account the biochemical properties including the nature of the catalytic residue, but also the oligomeric state of the proteins, and their tridimensional structure when solved (Frova, 2006; Mashiyama et al., 2014). The structural data notably showed that the N-terminal domain of soluble GSTs adopted a TRX fold, suggesting that the evolutionary history of soluble GSTs is linked to one of the TRX superfamily members. In this model, soluble GSTs were proposed to have evolved from a TRX/GRX ancestor to which a C-terminal helical domain has been added. Subsequent major transitions are the result of the dimerization of some GSTs, the replacement of the ancestral catalytic cysteine by a serine, and finally the change of this residue by a tyrosine in many mammalian GST classes. Although these major steps likely remain true, the current model is still incomplete, as it does not include the most recently identified classes such as mPGES2, GHR, Metaxin, Hemerythrin, Iota, and Ure2p, just to cite classes present in plants.

### Gene Organization and Distribution of Ser-GSTs in Eukaryote Photosynthetic Organisms

Among the 14 classes previously identified in terrestrial plants (Lallement et al., 2014), only five classes (Tau, Phi, Zeta, Theta, and TCHQD) clearly contain members possessing a conserved serine in their active site, even though this serine is absent in some isoforms. The DHAR, Hemerythrin, Iota, Lambda, GHR, mPGES2, and metaxin classes belong to the Cys-GSTs, as they primarily contain members possessing a conserved cysteine in their active site. For the EF1Bγ and Ure2p classes, the nature of the residue promoting GSH activation remains uncertain. Although this classification is based on the primary sequences, the recent release of several plant genomes allowed for its correlation with the intron-exon structure of GST-encoding genes as analyzed in *P. trichocarpa* (Lan et al., 2009), *P. patens* (Liu et al., 2013), *Capsella rubella* (He et al., 2016), *S. lycopersicum* (Islam et al., 2017), *Ipomoea batatas* (Ding et al., 2017), and *Brassica rapa* (Khan et al., 2018). Indeed, the number of exons is generally conserved for genes belonging to the same class, e.g., 1 or 2 for genes encoding GSTUs, 3 for GSTFs, 9 or 10 for GSTZs, 7 for GSTTs, and 2 for TCHQDs.

Hence, combining the gene structure analysis with protein motifs specific to GST classes, sequence alignments and phylogenetic trees provide a robust view of the Ser-GST gene copy number present in a given organism. A comparative genomic analysis was carried out using 39 sequenced photosynthetic organisms available in Phytozome database [version 12 (Goodstein et al., 2012)] including 3 chlorophytes, 1 bryophyte, 1 lycophyte, and 34 angiosperms; clearly extending previous genomic surveys (Table 1; Ding et al., 2017; Monticolo et al., 2017; Plomion et al., 2018). 1859 sequences were retrieved by BLASTp (Basic Local Alignment Search Tool) using *A. thaliana* GST sequences as queries and standard parameters. It is worth noting that chlorophytes but not terrestrial plants contain Tyr-GST isoforms (respectively, 6, 4, and 7 in *Chlamydomonas reinhardtii, Micromonas pusilla*, and *Volvox carteri*) also shared by animals. The presence of such isoforms likely compensates the absence or low number of Ser-GSTs in these organisms (1 isoform for both *C. reinhardtii* and *V. carteri*, 0 for *M. pusilla*). In the following paragraphs, we emphasize the major features of the different Ser-GST classes, i.e., gene content and protein sequence characteristics.

**Table 1:** Ser-GST gene content in sequenced chlorophytes and embryophytes.

	GSTU	GSTF	GSTT	GSTZ	TCHQD
**Chlorophyte**					
*Chlamydomonas reinhardtii* v5.5	0	0	1	0	0
*Volvox carteri* v2.1	0	0	1	0	0
*Micromonas pusilla* CCMP1545 v3.0	0	0	0 ^(∗)^	0	0
**Embryophyte**					
*Physcomitrella patens* v3.3	0 ^(∗)^	9	2	1	5 ^(∗)^
**Tracheophyte**					
*Selaginella moellendorffii* v1.0	38	1	3	2	1
**Angiosperm**					
*Amborella trichopoda* v1.0	22	4	1	2	1
**Grass**					
*Brachypodium distachyon* v3.1	40	21	1	3	1
*Oryza sativa* v7_JGI	45	16	1	4	1
**Panicoideae**					
*Setaria italica* v2.2	48	16	1	5	1
*Sorghum bicolor* v3.1.1	53	17	2	4	1
*Zea mays* Ensembl-18	34	10	2	2	1
**Eudicot**					
*Aquilegia coerulea* v3.1	24	29	3	2	1
**Pentapetalae**					
**Asterid**					
*Mimulus guttatus* v2.0	17	5	2	0 ^(∗)^	1
*Solanum lycopersicum* iTAG2.4	45	4	3	2	1
*Solanum tuberosum* v4.03	50	4	1	2	1
**Rosid**					
*Eucalyptus grandis* v2.0	62	19	1	2	7 ^(∗)^
*Vitis vinifera* Genoscope.12X	36	8	1	3	1
*Quercus robur*	62	12	1	2	1
**Poplar-Malvidae**					
**Malpighiales**					
*Linum usitatissimum* v1.0	30	11	4	3	2
*Manihot esculenta* v6.1	44	8	4	2	1
*Populus trichocarpa* v3.0	54	8	2	2	1
*Ricinus communis* v0.1	31	4	3	2	1
**SBM**					
**Citrus**					
*Citrus sinensis* v1.1	25	6	1	2	1
*Citrus clementina* v1.0	42	8	2	3	1
**Brassicales-Malvales**					
*Theobroma cacao* v1.1	36	9	1	2	1
**Brassicaceae**					
*Arabidopsis lyrata* v2.1	29	13	1	2	1
*Arabidopsis thaliana* TAIR10	28	13	3	2	1
*Boechera stricta* v1.2	27	12	1	1	1
*Brassica rapa* FPsc v1.3	38	20	2	2	1
*Capsella grandiflora* v1.1	21	10	1	2	1
*Capsella rubella* v1.0	26	12	1	1	1
*Eutrema salsugineum* v1.0	24	11	1	2	1
**Fabidae**					
**Nitrogen-fixing**					
*Cucumis sativus* v1.0	24	3	1	2	1
*Fragaria vesca* v1.1	28	5	1	2	1
*Glycine max* Wm82.a2.v1	50	10	3	3	2
*Malus domestica* v1.0	34	10	1	4	1
*Medicago truncatula* Mt4.0v1	47	10	2	2	1
*Phaseolus vulgaris* v2.1	24	12	2	2	2
*Prunus persica* v2.1	47	9	1	2	1


*Sequences have been retrieved from Phytozome v12.1, a Joint Genome Institute database. ^(∗)^ Unusually high/divergent gene copy number or absence of a given gene for a few specific organisms must be regarded with cautious as this may originate from bad genome assemblies or annotation problems or from remaining pseudogenes or gene alleles.*

#### GSTs Theta (GSTTs)

In addition to plants, GSTTs are also found in animals, insects, fungi, and bacteria; suggesting that this class appeared early during evolution (Coggan et al., 2002; Bryant et al., 2006; Skopelitou et al., 2012; Han et al., 2016; Shao et al., 2017). In photosynthetic organisms, the number of GSTT genes ranges from 1 to 4 (Table 1). According to its early appearance, this is the only Ser-GST class present in the chlorophytes (green algae) analyzed. Its absence in *M. pusilla* might be due to a gene loss event during evolution, unless there are annotation problems. In organisms having 2 or more *GSTT* genes, the genes are often organized in cluster such as in *A. thaliana* (Dixon et al., 2002), *Linum usitatissimum, Manihot esculenta* or *Ricinus communis* suggesting that tandem duplication(s) occurred during evolution from an ancestral gene. Whether the resulting proteins have diverged in function remains to be explored. The GSTT proteins are generally about 250 amino acids long. The conserved serine is found around position 10 in a conserved SQPS active site signature, which (with a few exceptions) is conserved among mammals (SQPC) and insects (S[Q/A]PC). At the subcellular level, these proteins have a peroxisomal localization, which is consistent with the presence of C-terminal SK[I/M] targeting motif (Dixon et al., 2009). Peroxisomes are multifunctional organelles involved notably in the β-oxidation of fatty acids in plants, a catabolic pathway contributing in particular to the production of acetyl-CoA, NADH, and FADH_2_, but also many lipid peroxides from polyunsaturated fatty acids that are the likely physiological substrates of GSTTs. *In vitro*, these enzymes exhibit a weak GSH-conjugation activity against conventional model substrates but they have a high GSH-dependent peroxidase activity toward linoleic acid peroxides (Dixon and Edwards, 2009).

#### GSTs Zeta (GSTZs)

In addition to plants, GSTZs are also present in bacteria, fungi, and animals, even though this is often as a reduced number of isoforms. A possible reason is their specific involvement in a general process, the tyrosine catabolism (Edwards et al., 2011). In photosynthetic organisms used for the present analysis (Table 1), the number of GSTZ genes ranges from 1 to 5 but we could not find them in chlorophytes. They are often found as tandem duplicates in genomes such as in *A. thaliana, C. rubella*, or *O. sativa*.

At the protein level, GSTZs are about 225 amino acids long. The serine is located around position 20 and is included in a conserved SSC(S/A) active site signature, the first serine being the residue necessary for the GSH-conjugation reaction. The catalytic mechanism of GSTZs differs from other GSTs in that GSH is initially conjugated to the *cis* double bond of maleylacetoacetate allowing the isomerisation reaction, before being eliminated in a second step allowing the formation of fumarylacetoacetate (Thom et al., 2001). Thus, it is assumed that the conserved cysteine performs the deglutathionylation of the intermediate product at the manner of Cys-GSTs or as proposed also for TCHQDs (see below). However, bacterial GSTZs lacking this cysteine catalyze the same reaction.

#### Tetrachlorohydroquinone Dehalogenases (TCHQDs)

TCHQDs have been identified in animals, fungi and plants. Plant genomes usually contain a single gene but 2, 5, and 7 TCHQD-encoding genes were identified in *Glycine max, P. patens*, and *Eucalyptus grandis*, respectively (Table 1). At the protein level, TCHQDs are on average 265 amino acids long. These proteins were first discovered in the soil bacterium *Sphingobium chlorophenolicum*, that is able to use pentachlorophenol, a fungicide used in wood preservation, as a carbon source. During the enzymatic degradation of pentachlorophenol, this bacterial TCHQD (PcpC) catalyzes the reductive dehalogenation of tetrachlorohydroquinone to trichlorohydroquinone and then to dichlorohydroquinone (Xun et al., 1992). PcpC possesses a peculiar SCIS signature containing both a serine and a cysteine. Accordingly, it reduces chloroquinones in two steps. The first step requires the serine in the GSH-conjugation of the quinone causing the departure of a chloride ion. The second step is the removal of the glutathione moiety from the quinone, a reaction performed by a nucleophilic attack of the cysteine (Willett and Copley, 1996; Kiefer and Copley, 2002). In plant proteins, there is no cysteine in the signature (often SLDS) (Lallement et al., 2014). They should therefore not be able to carry out deglutathionylation steps and may have different substrates or reaction mechanisms. Another possibility is that other GST isoforms such as GHRs substitute to TCHQDs as they are able to catalyze quinone deglutathionylation (Lallement et al., 2015).

#### GSTs Phi (GSTFs)

The *GSTF* genes are found in all terrestrial non-vascular or vascular plants that have been analyzed, but are absent in green algae/chlorophytes, suggesting important functions for terrestrial life. The gene content is very variable between species as it ranges from 1 in *Selaginella moellendorffii* to 29 in *Aquilegia coerulea* (Table 1). In almost all genomes, a large part of these genes is organized as clusters indicating repetitive, species-specific duplications.

At the protein level, GSTFs are about 215 amino acids long and the serine is located around position 12. Given the higher number of isoforms compared to the above-described classes, the four residue signature (reminiscent of the TRX/GRX family) is more variable. Most isoforms have the conserved serine but a few, exemplified by PtGSTF8 (AVCP), AtGSTF11 (AANP), or AtGSTF12/TT19 (AACP) are lacking it. In fact, the presence of the serine is not mandatory for the GSH-conjugation reaction as shown *in vitro* using poplar GSTFs (Pégeot et al., 2017). Several subgroups have been distinguished previously, according notably to this signature but also depending on the presence of N- or C-terminal extensions (Pégeot et al., 2014). Also, it was observed that some isoforms containing a cysteine exhibit a more diversified activity profile, as they possess deglutathionylation activity in addition to the peroxidase and GSH-conjugation activities (Pégeot et al., 2017).

#### GSTs Tau (GSTUs)

In light of current genomic resources, GSTUs form a plant specific class as is also the case for DHARs and GSTLs; two Cys-GST classes. Except in rare instances, such as in *Triticum aestivum* and *A. coerulea*, which contains respectively 38 and 29 GSTFs vs 26 and 24 GSTUs (Gallé et al., 2009), the GSTU class represents the largest GST class. From the absence of GSTU in green algae and in the mosses *P. patens* and *Sphagnum fallax*, the presence of only 2 GSTUs in another moss: *Marchantia polymorpha*, but the presence of 38 GSTUs in the bryophyte *S. moellendorffii*, we conclude that these genes have rapidly and dramatically expanded between bryophytes and lycophytes. They became predominant in vascular plants, being supposedly required for novel functions associated to the lifestyle of these plants. In angiosperms, the GSTU gene content is variable and range from 21 (*C. grandiflora*) to 62 (*Quercus robur* and *E. grandis*) (Table 1). The phylogenetic analysis of this family indicates that large clades are formed by proteins from the same species pointing to the fact that species-specific expansions occurred (Plomion et al., 2018). They normally correspond to genomic clusters produced by several successive tandem duplication events as exemplified in poplar, *A. thaliana* or *O. sativa* (Wagner et al., 2002; Soranzo et al., 2004; Lan et al., 2009). Accordingly, it is difficult to define strict orthologs for a given isoform among the different species and to determine what is the set of GSTU ancestors shared by angiosperms.

Overall, the GSTU and GSTF classes represent around 75% of all GST genes as in *Q. robur* (62 out of 88 genes) (Plomion et al., 2018) or *A. thaliana* (41 out of 55 genes) (Dixon and Edwards, 2010a). For both classes, the expansion, specific genomic organization and high sequence similarity among duplicated members have important implications. One consequence may be the existence of functional redundancy between isoforms making it difficult to study the biological functions of a particular gene using reverse-genetic approaches. However, another consequence may be that upon duplication, some of the new gene copies, which have been less subject to evolutionary pressure and have accumulated mutations, have likely acquired structural and functional diversity. The truth is certainly in between and this remains to be experimentally addressed in a more exhaustive manner.

### Biochemical Properties and Catalytic Activities of Ser-GSTs

GSTs are versatile enzymes, accommodating diverse substrates/ligands in the active site or L-sites (Table 2), and catalyzing diverse enzymatic reactions as a function of the active site signature (Chronopoulou et al., 2017a). Besides the so-called ligandin function, Ser-GSTs catalyze GSH-conjugation reactions on numerous types of substrates, the reduction of organic hydroperoxides or substrate isomerisation whereas Cys-GSTs rather catalyze opposite reactions including the reduction of glutathione conjugates. For the latter aspect, we invite the reader to refer to the recent review describing Cys-GST properties (Lallement et al., 2014). For all the catalytic activities, the high reactivity of a cysteine residue (either from GSH or from the polypeptide) plays a central role in the biochemical properties carried out by GSTs. Noteworthy, some Ser-GSTs belonging to the GSTZ, TCHQD, or GSTF classes possess a cysteine residue in the catalytic center, which confers them dual activity profile.

**Table 2:** Ligands of plant Ser-GSTs identified.

Isoform	Organism	Ligands	References
Bronze-2 or Bz2 (GSTU)	*Zea mays*	Cyanidin-3-glucoside	Marrs et al., 1995
ZmGSTI-I, ZmGSTI-II, ZmGST II-II, ZmGST III-III (GSTU et GSTF)	*Zea mays*	Protoporphyrin IX, mesoporphyrin, coproporphyrin, uroporphyrin, Mg-protoporphyrin	Lederer and Böger, 2003
ZmGSTU1	*Zea mays*	Uroporphyrin, pentacarboxyl porphyrin, harderoporphyrin-SG, coproporphyrin, heme B	Dixon et al., 2008
ZmGSTF1	*Zea mays*	Gibberellic acid, indole-3-butyric acid, 2-naphtoxyacetic acid, 2,4-dichlorophenoxyacetic acid, kinetin, quercetin, luteolin	Axarli et al., 2004
AtGSTU7	*Arabidopsis thaliana*	Protoporphyrin-SG, myristoyl-glutathione	Dixon and Edwards, 2009, 2018
AtGSTU19	*Arabidopsis thaliana*	Harderoporphyrin-SG, chlorogenic acid, 10-S-glutathionyl-12-oxo-phytodienoic acid, oxylipin-SG, 3-methylindolyl glutathionyl disulfide, 12-oxo-phytodienoic acid (OPDA)	Dixon and Edwards, 2009, 2018
Transparent testa 19 or TT19 (GSTF)	*Arabidopsis thaliana*	Anthocyanin	Kitamura et al., 2004
AtGSTF2, AtGSTF3	*Arabidopsis thaliana*	Norharmane, harmane, lumichrome, indole-3-aldehyde, quercetin-3-O-rhamnoside	Dixon et al., 2011a
AtGSTF2	*Arabidopsis thaliana*	Indole-3-aldehyde, camalexin, quercetrin, quercetin	Ahmad et al., 2017
AtGSTF2	*Arabidopsis thaliana*	Grossamide K-SG, cannabisin, 10-S-glutathionyl-12-oxo-phytodienoic acid, kaempferol-3,7,4′-trimethylether, quercetin-3,7,3′,4′-tetramethylether	Dixon and Edwards, 2018
Anthocyanin9 or An9 (GSTF)	*Petunia hybrida*	Anthocyanin	Alfenito, 1998
Anthocyanin9 or An9 (GSTF)	*Petunia hybrida*	Isoquercitrin, quercetin, cyanidin, luteolin	Mueller et al., 2000
GST	*Hyoscyamus muticus*	Indole-3-acetic acid	Bilang et al., 1993
CkmGST3 (GSTF)	*Cyclamen persicum* ×*Cyclamen purpurascen*s	Anthocyanin	Kitamura et al., 2012
VvGST1 (GSTU), VvGST4 (GSTF)	*Vitis vinifera*	Anthocyanin	Conn et al., 2008
VvGSTU2	*Vitis vinifera*	*Trans-*resveratrol	Martínez-Márquez et al., 2017
Flavonoid3 or Fl3	*Dianthus caryophyllus*	Anthocyanin	Larsen et al., 2003
PfGST1 (GSTF)	*Perilla frutescens*	Anthocyanin	Yamazaki et al., 2008
AtGSTU9, AtGSTU10	*Arabidopsis thaliana*	fatty acyl (C14,C16,C17, C18)	Dixon and Edwards, 2009
AtGSTU25, AtGSTU28	*Arabidopsis thaliana*	fatty acyl (C6,C8,C10,C12,C14)	Dixon and Edwards, 2009
AtGSTF6	*Arabidopsis thaliana*	Indole-3-acetonitrile	Su et al., 2011
AtGSTU13	*Arabidopsis thaliana*	Indole-3-ylmethyl-ITC (indole glucosinolate)	Piślewska-Bednarek et al., 2018


#### GSH-Conjugating Activity

Most Ser-GSTs catalyze the conjugation of GSH onto electrophilic compounds including aromatic, aliphatic or heterocyclic compounds (Deponte, 2013). This conjugation requires the binding of a GSH molecule in the glutathione binding site (G-site). In most cases, the presence of a specific residue, most often a cysteine, serine, or tyrosine at the vicinity of the cysteine of the bound GSH, induces a decrease in the thiol pKa (Board and Menon, 2013; Deponte, 2013). This pKa, usually around 9, is lowered to approximately 6.5 or even less, promoting the formation of a nucleophilic (reactive) thiolate group *in vivo*, which is able to perform a nucleophilic attack on a nearby electrophilic substrate. In other words, the conserved serine in the active site of Ser-GSTs does not play the role of the catalytic residue; rather, this is carried out by the thiolate group of GSH. The nucleophilic attack initiated by GSH occurs either by a substitution (e.g., on a chlorine atom) or by the reduction of an electron acceptor via a Michael’s addition (Deponte, 2013). Several model substrates are used to measure GSH-conjugation activity, the most common being the 1-chloro-2,4-dinitrobenzene (CDNB). The detoxification of herbicides (e.g., atrazine and fluorodifen) and pesticides (e.g., alachlor and metolachlor) through GSH-conjugation has been extensively studied over the years, notably using GSTs from crops (Gronwald and Plaisance, 1998; Cummins et al., 2003; Cho et al., 2007).

#### Peroxidase Activity

In the cells, hydroperoxides are reduced by several families of enzymes, in particular thiol peroxidases including the peroxiredoxin (PRX) and glutathione peroxidase-like protein (GPXL) families (Rouhier and Jacquot, 2005). Some GSTs also exhibit peroxidase activity, as they are able to reduce (hydro)peroxides into alcohols concomitant to the oxidation of GSH into GSSG. In this reaction, deprotonated GSH bound to the G-site of the enzyme induces a nucleophilic substitution of the bond between the two oxygen atoms of the hydroperoxyl group (R-OOH), leading to the release of GSOR and an OH group that is protonated into H_2_O. GSOR is then cleaved into GSSG and ROH by a second nucleophilic substitution by the GSH molecule according to a mechanism that is not yet fully elucidated (Deponte, 2013). The GSTs performing this reaction have catalytic efficiencies (10^2^ to 10^4^ M^-1^⋅s^-1^) measured in steady-state conditions in the range of those of TRX- and GRX-dependent thiol peroxidases (Pégeot et al., 2017). A major difference is, however, the rate of the first step (i.e., peroxide reduction) that occurs at turnover numbers up to 10^7^ s^-1^ for some thiol peroxidases. The contribution of GSTs in the reduction of cellular hydroperoxides remains poorly described but it might be important because these are almost the only GSH-dependent peroxidases, most thiol peroxidases being dependent on GRXs or TRXs, including GPXLs (Rouhier and Jacquot, 2005; Navrot et al., 2006). Also, their contribution appears to be different depending on the organisms and the subcellular compartments considered. Among Ser-GSTs, only those belonging to the Phi, Tau, and Theta classes are able to catalyze such reactions, peroxisomal GSTTs being likely specialized in the reduction of fatty acid peroxides (Dixon et al., 2009).

#### Isomerase Activity

Some GSTs, in particular from the Zeta class, catalyze the GSH-dependent isomerisation of specific metabolites, such as the *cis-trans* isomerisation of maleylacetoacetate into fumarylacetoacetate occurring during the penultimate step of tyrosine catabolism in eukaryotes (Fernández-Cañón and Peñalva, 1998; Thom et al., 2001; Fernandez-Canon et al., 2002; Edwards et al., 2011). In some bacteria, GSTZs function as maleylpyruvate isomerases (Marsh et al., 2008) and catalyze the isomerisation of maleylpyruvate into fumarylpyruvate through the third committed step in the degradation of salicylate to the metabolites pyruvate and fumarate via gentisate. Contrary to other activities described above, GSH is not considered as a substrate but as a cofactor because it is not consumed during the reaction (Litwack et al., 1971; Marsh et al., 2008).

#### Non-enzymatic Binding and Intracellular Transport

In addition to their catalytic function, GSTs also serve as non-enzymatic carrier proteins or ligandins (Habig et al., 1974; Mannervik and Danielson, 1988). The term “ligandin” was historically associated to proteins characterized in animals, which were able to bind a wide range of hydrophobic ligands, such as steroids, heme and its degradation product bilirubin, carcinogens, and bile salts. These proteins turned out to be GSTs from the Alpha and Mu classes (Levi and Arias, 1969; Ketley et al., 1975). Since then, plant GSTUs and GSTFs were found to bind several tetrapyrroles, e.g., protoporphyrin IX (Proto IX), Mg-protoporphyrin but also bacterial porphyrin derivatives identified upon expression of recombinant maize GSTUs (Lederer and Böger, 2005; Dixon et al., 2008). However, it is not clear whether this is strictly a ligandin function as *Zea mays* GSTU1 is able to catalyze the conjugation of GSH on protoporphyrinogen IX and harderoporphyrinogen (Dixon et al., 2008). Another example, likely the best described, concerns anthocyanins, which are phenolic antioxidant compounds conferring the colors we commonly associate with fruits and flowers. These molecules are transiently bound/transported by GSTs before their release to ABC-type transporters for vacuolar sequestration. This is documented for both Tau and Phi class members, i.e., Bz2 (GSTU) from maize, AN9, TT19, and VvGST4 (GSTFs) from *Petunia hybrida, A. thaliana* and *Vitis vinifera*, respectively (Marrs, 1996; Alfenito, 1998; Mueller et al., 2000; Kitamura et al., 2004; Conn et al., 2008; Gomez et al., 2011; Momose et al., 2013; Zhao, 2015). Other molecules were isolated using ligand fishing approaches (Table 2). GSTU2 from *V. vinifera* binds *trans-*resveratrol, a polyphenol transported from the cells into extracellular medium and conferring antimicrobial properties (Martínez-Márquez et al., 2017). GSTF2 and GSTF3 from *A. thaliana* were described *in vitro* to bind various ligands such as lumichrome, harmane, norharmane, indole-3-aldehyde, camalexin, and quercetin-3-O-rhamnoside (Dixon et al., 2011a) but the physiological significance remains unknown. In addition to a carrier function, it may be that the non-enzymatic binding of molecules prevents their oxidative damage, thus ensuring a protective effect (Mueller et al., 2000).

### Structural Characteristics of Plant Ser-GSTs

**FIGURE 1:**
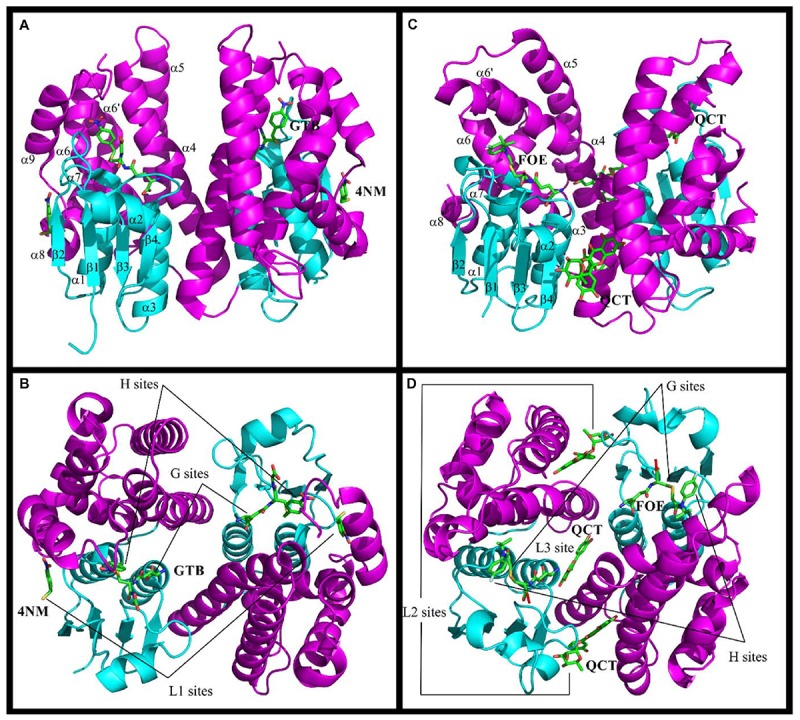
Structures of Ser-GSTs from plants highlighting the location of ligand-binding sites. **(A–D)** schematic structure of the GmGSTU4 and AtGSTF2 dimers, respectively. **(C,D)** illustrate the complexes formed between AtGSTF2 and FOE (1BX9) or QCT (5A4W). The secondary structures and the location of the ligand-binding sites are labeled. The TRX domain is in cyan and the C-terminal domain is in magenta. The labeled ligands are: GTB, S-(P-Nitrobenzyl)glutathione; 4NM, 4-Nitrophenyl methanethiol; FOE, FOE-4053-glutathione conjugate; QCT, Quercetrin.

Considering the high number of Ser-GSTs in plants, only a few structures have been solved so far: 27 for GSTUs, 15 for GSTFs, 1 for GSTZ, and none for TCHQD and GSTT (Table 3). All these plant Ser-GSTs are homodimeric enzymes in which each protomer of approximately 23–30 kDa contains two domains with a catalytic center at the interface. The N-terminal domain adopts the typical TRX-fold (with β1α1β2α2β3β4α3 topology) and the C-terminal domain is a bundle of at least five helices (α4 to α8) (Figure 1). GSTUs have an additional α9 helix that is oriented toward the active site without occluding it (Thom et al., 2002). In GSTFs, the α6-α7 connection systematically includes a small helix (α6′) (Reinemer et al., 1996; Pégeot et al., 2014, 2017). The atomic model of the only GSTZ crystal structure, that of *A. thaliana* GSTZ1, is incomplete between helices α4 and α5, which hinders the accurate determination of the active site (Thom et al., 2001). Within a class, the variable regions are often close to the active site and involved in the binding of the electrophilic substrate. In GSTUs, these regions include helix α9 and the segment from roughly the C-terminal end of α4 to the N-terminal end of α5 (Valenzuela-Chavira et al., 2017). In GSTFs, they include this segment and the connection β2-β3, which, in maize GSTF3, was supposed to move upon binding of the substrate in the active site (Neuefeind et al., 1997a). This connection is also involved in the dimer stabilization (see below).

**Table 3:** Crystal structures of Ser-GSTs from plants.

Class	Organism	Name	Ligand	PDB Entry	References
	*Arabidopsis thaliana*	AtGSTF2	GTX^a^ (1GNW)^b^, FOE^a^ (1BX9)^b^, 7WB^a^ (5A5K)^b^, QUE^a^ (5A4V)^b^, I3A^a^ (5A4U)^b^, QCT^a^ (5A4W)^b^	1GNW, 1BX9, 5A5K, 5A4V, 5A4U, 5A4W	Reinemer et al., 1996; Prade et al., 1998; Ahmad et al., 2017
**Phi**	*Populus trichocarpa*	PtGSTF1	GSH^a^ (4RI6)^b^, GSH^a^ (4RI7)^b^	4RI6, 4RI7^c^	Pégeot et al., 2014
		PtGSTF2		5EY6	Pégeot et al., 2017
		PtGSTF5	GSH^a^	5F05	
		PtGSTF7	GSH^a^	5F06	
		PtGSTF8	GSH^a^	5F07	
	*Zea mays*	ZmGSTF1	CYW^a^ (1AXD)^b^, ATA^a^ (1BYE)^b^	1AXD, 1BYE	Neuefeind et al., 1997a; Prade et al., 1998
		ZmGSTF3		1AW9	Neuefeind et al., 1997b
	*Triticum aestivum*	TaGSTU4-4	GTX^a^	1GWC	Thom et al., 2002
	*Arabidopsis thaliana*	AtGSTU20/ FIP1	GSH^a^	5ECS, 5ECR, 5ECQ, 5ECP, 5ECO, 5ECN, 5ECM, 5ECL, 5ECK, 5ECI, 5ECH	Chen et al., 2017
		AtGSTU23	GSH^a^ (6EP7)^b^	6EP6, 6EP7, 5O84	Tossounian et al., 2018^d^
		AtGSTU25	GSSG	5G5A	^*d*^
**Tau**	*Glycine max*	GmGSTU4	GTB^a^ (2VO4, 5AGY)^b^, GSH^a^ (4TOP)^b^	2VO4, 4TOP, 5AGY^e^	Axarli et al., 2009a,b; Burmeister et al., 2008
		GmGSTU10-10	GS8^a^	4CHS	Skopelitou et al., 2015
	*Mangifera indica*	MiGSTU	GSH^a^ (5G5F), GTX^a^ (5KEJ)	5G5E, 5G5F, 5KEJ	Valenzuela-Chavira et al., 2017
	*Oryza sativa subsp. japonica*	OsGSTU1	GSH^a^	1OYJ	Dixon et al., 2003
	*Populus trichocarpa*	PtGSTU30	GSH^a^	5J4U, 5J5N^f^	Yang et al., 2019^d^
	*Ricinus communis*	EFI-501866		4J2F	^*d*^
**Zeta**	*Arabidopsis thaliana*	GSTZ1		1E6B	Thom et al., 2001


*^*a*^PDB ligand codes: GSH, Glutathione; GTX, S-hexylglutathione; FOE, FOE-4053-glutathione conjugate; 7WB, Camalexin; QUE, Quercetin; I3A, Indole-3-aldehyde; QCT, Quercetrin; CYW, Lactoylglutathione; ATA, Atrazine glutathione conjugate; GTB, S-(P-Nitrobenzyl)glutathione; GS8, S-Hydroxy-glutathione. ^*b*^PDB, entry where the ligand is present. ^*c*^4RI7: crystal structure of an isoform of PtGSTF1, which contains mutation of S13 to C. ^*d*^crystal structures only available in the PDB. ^*e*^5AGY: crystal structure of an isoform of GmGSTU4, which contains mutations of I183 to V, Q46 to K, R38 to Q, and W114 to C. ^*f*^5J5N: crystal structure of an isoform of PtGSTU30, which contains mutation of R39 to W.*

Concerning dimerization, the GSTU dimer has an open V-shaped configuration with 2200 Å^2^ of the accessible surface that is buried at the interface, comparable with that of GSTOs (2000 Å^2^), but smaller than that of GSTFs (2700 Å^2^) and most other classes of GSTs (2800–3400 Å^2^) (Axarli et al., 2010). The monomers are related by a two-fold symmetry where the N-terminal domain of one subunit cross-interacts with the C-terminal domain of the second one, and vice versa. The contact regions are the loop α2-β2, the strand β3 and the helix α3 of one monomer and the helices α4 and α5 of the other. The dimerization interface involves hydrophobic surface patches and a particular lock-and-key motif in which the side-chain of an aliphatic or aromatic residue extends across the dimer interface (Val52 in TaGSTU4 and Phe53 in PtGSTF1, Table 3). In GSTUs, conserved salt bridges close to the dyad axis bind both subunits. In GSTFs, the number and the nature of the polar interactions vary significantly from one isoform to another. Indeed, a single hydrogen bond connects the two subunits of AtGSTF2 (Reinemer et al., 1996) whereas nine are found in PtGSTF1 (Pégeot et al., 2014).

**FIGURE 2:**
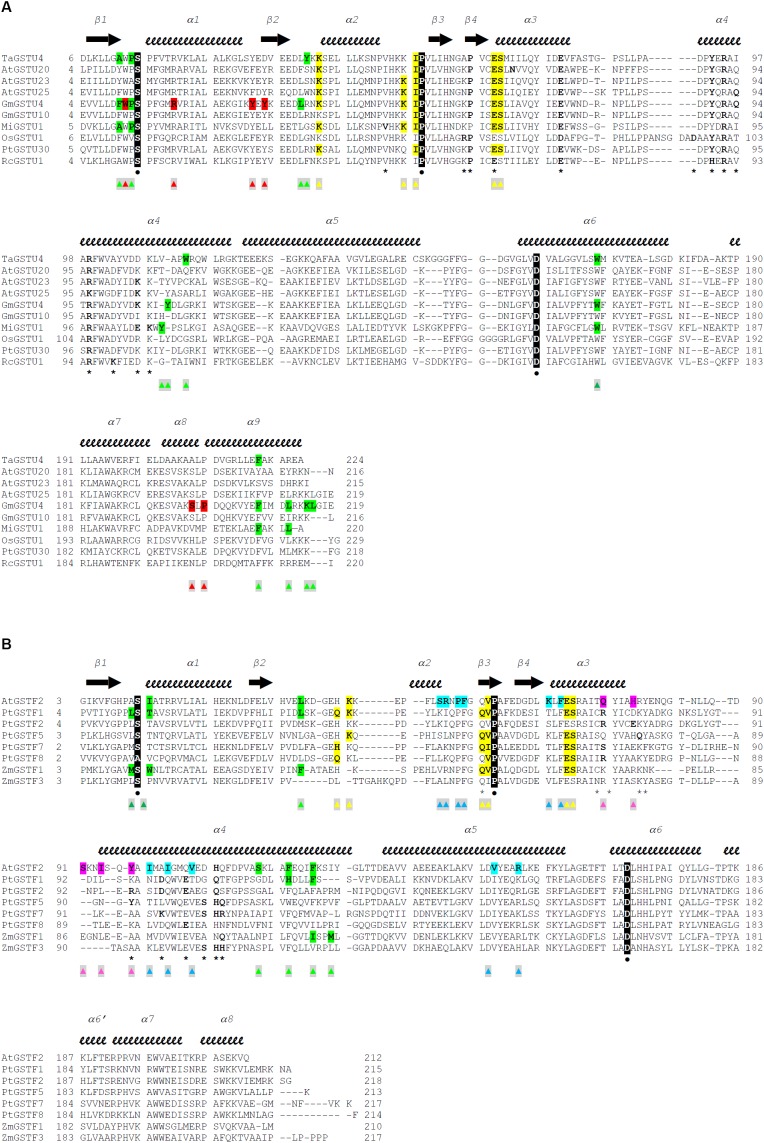
Structure-based sequence alignments of Tau class **(A)** and Phi class **(B)** GSTs from plants. The sequence alignment was generated with Chimera (Pettersen et al., 2004) and manually adjusted. Crystal structures and sequences are available at the Protein Data Bank (http://www.rcsb.org): 1GWC for TaGSTU4, 5ECS for AtGSTU20, 6E6P for AtGSTU23, 5G5A for AtGSTU25, 2VO4 for GmGSTU4, 4CHS for GmGSTU10, 5G5E for MiGSTU1, 1OYJ for OsGSTU1, 5J4U for PtGSTU30, 4J2F for RcGSTU1, 1GNW for AtGSTF2, 4RI6 for PtGSTF1, 5EY6 for PtGSTF2, 5F05 for PtGSTF5, 5F06 for PtGSTF7, 5F07 for PtGSTF8, 1AXD for ZmGSTF1, and 1AW9 for ZmGSTF3. Secondary structures are labeled and shown using arrows (β-strands) and squiggles (helices). The active site serine, the invariant proline and the quasi-invariant aspartic acid are in bold type, colored white, highlighted black, and marked with 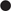. Residues that participate in dimer stabilization via strong polar interactions are in bold and marked with **^∗^**. Residues involved in binding glutathione (G-site) are in bold type, highlighted yellow, and marked with 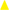. Residues of the characterized H-sites are in bold type, highlighted green, and marked with 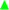. Residues of the L1-site (GmGSTU4, 2VO4) are in bold type, highlighted red, and marked with 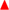. Residues of the L2-site (AtGSTF2, 5A4U, 5A4V, and 5A4W) are in bold type, highlighted blue, and marked with 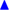. Residues of the L3-site (AtGSTF2, 5A4K, 5A4U, and 5A4W) are in bold type, highlighted pink, and marked with 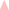.

The GST catalytic center is usually divided in two distinct functional regions, a hydrophilic G-site for binding glutathione, and an adjacent hydrophobic H-site for accommodating electrophilic substrates. The anchoring residues of the G-site are well conserved among all GSTs probably because of their high specificity for glutathione. These residues are highlighted in the structural alignments (Figure 2). In Ser-GSTs, the GSH thiol group is normally hydrogen bonded to the hydroxyl group of the catalytic serine (Ser13 in PtGSTF1) (Pégeot et al., 2014). However, this serine is important but not mandatory for GSH-conjugating reactions as concluded from mutagenesis studies or its absence in some GSTFs (Pégeot et al., 2014). In poplar GSTFs, nearby hydroxylated residues present in the active site signature (often STxT) could be involved in GSH activation (Pégeot et al., 2017). Generally speaking, the H-site is built from elements from both the N- and C-terminal domains. The observed variations reflect the broad electrophilic-substrate specificities of the different GST isoforms/classes. Only AtGSTF2 and ZmGSTF1 crystal structures were obtained in the presence of herbicidal-glutathione conjugates (Figure 1; Prade et al., 1998). In other cases, the H-sites have been defined from the presence of inhibitors such as S-hexylglutathione or molecules from the crystallization medium. In the large majority of cases, the putative H-site residues are hydrophobic in nature. In GSTFs, the H-site involves residues located around the catalytic serine (N-terminal end of helix α1), in the loop β2-α2 and in the C-terminal end of helix α4. In GSTUs, residues from two additional regions are concerned, namely the helix α6 and the additional helix α9. A conserved tryptophan is present in the helix α6 of GSTUs (Trp171 in TaGSTU4) (Thom et al., 2002). In GSTFs, aromatic residues identified by mutagenesis studies have been clearly demonstrated as participating in the affinity toward electrophilic substrates (Axarli et al., 2004; Dixon et al., 2011a).

The structures of GSTs include other important regions that are associated with non-catalytic functions. AtGSTU20, also named FIP1 (FIN219-interacting protein 1) because it interacts with the jasmonate-amido synthetase FIN219, participates in the jasmonate signaling response under far-red light conditions (Chen et al., 2007, 2017). In a crystallographic study, it was shown that the formation of the FIP1-FIN219 complex results in the reorientation of the FIN219 C-terminal domain, which appears crucial for improving jasmonoyl-isoleucine biosynthesis. However, the overall conformation of AtGSTU20 is not altered and the FIN219-binding region includes the C-terminal α6 to α8 helices. Interestingly, the authors noted that some of the contact residues are well conserved in GSTUs and GSTs of other species (Figure 2; Chen et al., 2017). For their ligandin function, GSTs bind a wide range of compounds in a non-catalytic manner at so-called L-sites, which are often distinct from the active site. Three different L-sites were described in GSTs from plants (Figure 1). The structural analysis of GmGSTU4-4 revealed the presence of one molecule of (4-nitrophenyl)methanethiol in each subunit in a hydrophobic surface pocket (L1-site) (Axarli et al., 2016). The bottom and walls of the L1-site are lined with residues from α1, β2, and α8. The main binding residues are conserved in GSTUs. The crystal structures of AtGSTF2 in complex with two indole derivatives and two flavonoids revealed two other ligand-binding sites (L2 and L3) (Ahmad et al., 2017) extending the observation of tight protein-ligand interactions (*K*_d_ < 1 μM) by isothermal titration calorimetry (Dixon et al., 2011a). The L2 site is situated between helices α4 and α7 in each monomer whereas the L3 site is located at the base of the dimer interface involving helices α3 of one subunit and α4 of its neighbor (Figure 1). All ligands are stabilized mainly through hydrophobic interactions (Ahmad et al., 2017). Coupled to biochemical evidence, the presence of these non-catalytic L-sites in GSTUs and GSTFs suggest that at least some of them should function in the transport of endogenous metabolites (Dixon et al., 2011a). However, the residues forming these L-sites are difficult to identify because they are not well-conserved among plant GSTs (Ahmad et al., 2017).

### Gene Expression of Ser-GSTs in *A. thaliana*

Analyzing the transcript abundance of these GSTs could be helpful to understand the possible redundancy between close/duplicated isoforms as well as to give clues about their functions in the absence of molecular and genetic information. In fact, it is quite well documented in many species that the expression of *GSTF* and *GSTU* genes is often induced in response to environmental constraints. This includes heavy-metal exposure (Moons, 2003; Ahsan et al., 2008; Reid et al., 2013; Tripathi et al., 2014), salinity, heat, cold, drought (Jha et al., 2011; Tiwari et al., 2016; Yang et al., 2016; Xu et al., 2018; Srivastava et al., 2019), or biotic interactions such as pathogenic interaction (Rinaldi et al., 2007; Skopelitou et al., 2015; Kao et al., 2016). The expression of several *GSTF* and *GSTU* genes is also enhanced in response to phytohormones including abscisic acid, auxin, ethylene, methyl jasmonate and salicylic acid, to herbicides and to herbicide safeners, and more generally to treatments leading to an oxidative stress (DeRidder, 2002; Wagner et al., 2002; Lieberherr et al., 2003; Smith et al., 2003, 2004; Sappl et al., 2004, 2009; Chen et al., 2012; Chronopoulou et al., 2017b). Thus, using *A. thaliana* as a representative organism, the transcript abundance of 44 out of the 47 Ser-GST genes was retrieved from the AtGenExpress datasets. The expression profiles of GSTF2 (At4g02520), GSTF7 (At1g02920), and TCHQD (At1g77290) were not available and are therefore not present in this gene expression analysis.

**FIGURE 3:**
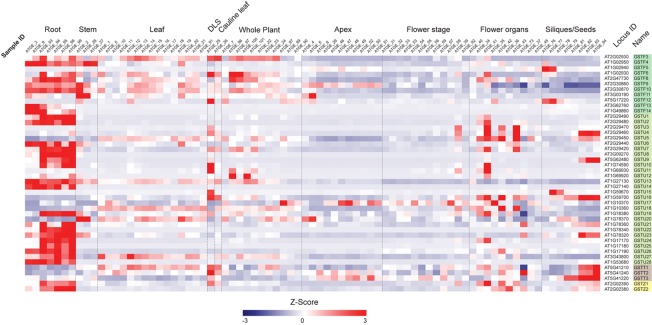
Transcript abundance of 44 Ser-GST genes during Arabidopsis development. Microarray experimental data (generated using Affymetrix ATH1 GeneChip arrays) of Arabidopsis Development (AtGenExpress Developmental Expression Atlas) described by Schmid et al. (2005) were obtained from the National Center for Biotechnology Information (NCBI) Gene Expression Omnibus (http://www.ncbi.nlm.nih.gov/geo/). Intensity values of replicates were averaged and z-score transformed across the following ten developmental conditions: roots, stems, rosette leaves, developmental leaf senescence (DLS), cauline leaf, whole plant, apex, flowering stages, flower organs, and siliques/seeds. The data were then imported into The Institute for Genomic Research Multiple Experiment Viewer (MVE) and hierarchically clustered using average linkage based on Euclidean distance. Gene families of each gene are indicated using the following color key: tau, light green; phi, medium green; theta, gray; and zeta, yellow.

First, we examined the expression profiles of Ser-GST genes in the context of a developmental time-course in Arabidopsis (Figure 3), using the AtGenExpress Developmental Set (Schmid et al., 2005). In this case, the transcript abundance of each gene was standardized using z-score transformation (a form of normalization that is particularly useful when comparing samples from diverse treatments/tissue backgrounds), and arranged by classes. One can clearly see that most GSTFs and GSTUs, as well as the two GSTZs, have their highest expression in roots. However, for the three GSTTs, the highest expression is found in samples spanning seed development (siliques/seeds), particularly in those containing isolated maturing seeds. In addition, these GSTTs also exhibited increased transcript abundance during developmental leaf senescence (DLS), however, this cannot be relied upon too strongly as only one time point is included in this dataset. Interestingly, within the GSTF family, *GSTF5* is only highly expressed at the two first stages of siliques/seeds (siliques bearing developing seeds), a specificity that is also found for *GSTF12*, albeit the transcript abundance also peaks during DLS. The *GSTF3, 6, 8, 9, 10*, and *11* genes are also expressed in leaves and in whole plant stages, but to a much lower level than in roots. As already said, most of the *GSTUs* display their highest expression levels in roots, however, *GSTU4, 9, 15*, and *16* are exclusively and strongly expressed at the siliques/seeds stages. An exception is *GSTU23*, which exhibits its highest transcript abundance in both roots and siliques/seeds stages. Finally, many Ser-GSTs appear to have transient expression during whole plant, leaf, flower, and stem development, which could suggest very specific functions in response to developmental cues.

**FIGURE 4:**
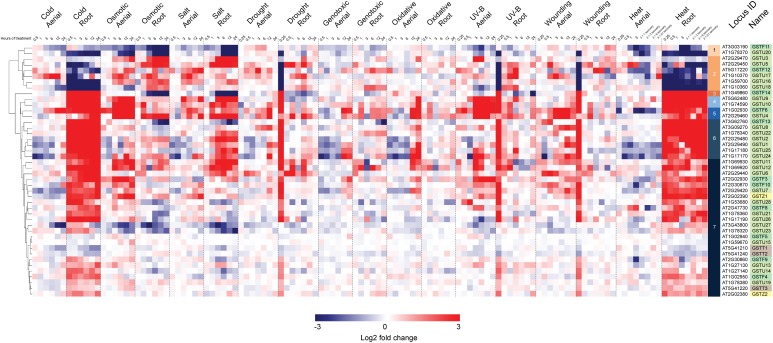
Hierarchical clustering of log2 fold changes of 44 Ser-GST genes in response to abiotic stresses. Microarray experimental data (generated using Affymetrix ATH1 GeneChip arrays), described by Kilian et al. (2007), were obtained from the NCBI Gene Expression Omnibus (http://www.ncbi.nlm.nih.gov/geo/). Intensity values of replicates were averaged and z-score transformed across nine stresses (cold, osmotic, salt, drought, genotoxic, UV, wounding, and heat) grouped as either aerial or root tissue, and further defined according to a time course of exposure in hours. Hierarchical clustering was carried out by average linkage based on Euclidean distance using in the Multiple Experiment Viewer (MEV) analysis package, resulting in the defining of 7 clusters. Gene families of each gene was indicated using the following color key: tau, light green; phi, medium green; theta, gray; and zeta, yellow.

In a second stage of analysis, we examined the expression profile of these same 44 Ser-GSTs in response to a number of abiotic stresses [AtGenExpress Stress Set; (Kilian et al., 2007)]. Data are presented as a log2 fold-change of the stress treatment (at a given time point) versus its respective control sample, and the entire dataset is hierarchically clustered using Euclidean distance (Figure 4). This analysis clearly demonstrates that most Ser-GSTs strongly respond to stresses applied to the roots and thus, substantiates the high expression profiles observed in roots from the developmental stage analysis (Figure 3). Interestingly, GSTs present in clusters 3, 4, 5, 6, and 7 exhibit high transcript abundance in response to temperature changes in roots (cold and heat). That said, the five GSTs comprising clusters 3, 4, and 5 additionally exhibit high transcript abundance in response to almost all stresses, in both aerial and subterranean tissues. Also of note here, whereas the 2 GSTs of cluster 1, (*GSTF11* and *GSTU20*) have an overall low fold-change of their transcript abundance in response to all stresses as compared to most of the other GSTs, the 6 GSTs included in cluster 2 seem to consistently respond to osmotic changes, salt and drought stresses in both root and aerial parts. Altogether, this clearly indicates that Ser-GSTs are involved in the molecular responses to several environmental cues, both biotic and abiotic, a fact that is discussed further in the next chapter.

### Physiological Roles of Ser-GSTs in *A. thaliana*

As highlighted above, Ser-GSTs constitute the largest group of GSTs in plants. Although several molecular and biochemical studies have shed light on their tridimensional structures, biochemical properties, and enzymatic activities, very little is known about the actual roles that these proteins play *in planta*. This lack of knowledge might reflect the functional redundancy that most probably exists between these GSTs (Sappl et al., 2009; Rahantaniaina et al., 2017). Nevertheless, the use of the model plant *A. thaliana* has aided in deciphering the role played by some of the 47 Ser-GSTs (13 GSTFs, 28 GSTUs, 1 TCHQD, 2 GSTZs, and 3 GSTTs) (Table 4; Wagner et al., 2002; Dixon and Edwards, 2010a).

**Table 4:** Diversity of Ser-GST functions.

GST	Gene ID	Acronym	Known role	Mutant characterized	References
GSTU1	At2g29490	GST19		None	
GSTU2	At2g29480	GST20		None	
GSTU3	At2g29470	GST21		None	
GSTU4	At2g29460	GST22		None	
GSTU5	At2g29450	GSTU1, AT103-1A	UV radiation acclimation	GSTU5-OE	Liu and Li, 2002
			Excess light acclimation response	None	Lv et al., 2015
GSTU6	At2g29440	GST24		None	
GSTU7	At2g29420	GST25	Part of the lipid stress response	None	Stotz et al., 2013
GSTU8	At3g09270			None	
GSTU9	At5g62480	GST14, GST14B	Salt and drought stress response	None	Ambrosone et al., 2015
GSTU10	At1g74590			None	
GSTU11	At1g69930			None	
GSTU12	At1g69920			None	
GSTU13	At1g27130	GST12	Indole glucosinolate biosynthesis / Response to fungal pathogens (*E. pisi, C. gloeosporioides* and *P. cucumerin*)	*gstu13*	Piślewska-Bednarek et al., 2018
GSTU14	At1g27140	GST13	Part of the excess light acclimation response	None	Lv et al., 2015
GSTU15	At1g59670			None	
GSTU16	At1g59700		Part of the plant response to fungal pathogens (*V. dahliae*)	None	Pantelides et al., 2010
GSTU17	At1g10370	ERD9, GST30, GST30B	Drought and salt stress response	GSTU17-OE and *gstu17*	Chen et al., 2012
			Light response / Seedling development / Root elongation	GSTU17-OE and *gstu17*	Jiang et al., 2010
GSTU18	At1g10360	GST29	Part of the lipid stress response	None	Mueller et al., 2008
GSTU19	At1g78380	GST8	Drought, salt and methyl viologen stress response	GSTU19-OE	Xu et al., 2016
GSTU20	At1g78370	FIP1	Light response / Seedling development / Flowering time	GSTU20-OE and *gstu20*	Chen et al., 2007
GSTU21	At1g78360			None	
GSTU22	At1g78340			None	
GSTU23	At1g78320			None	
GSTU24	At1g17170	GST	Lipid stress response	None	Mueller et al., 2008
			Xenobiotic detoxification (TNT, 2,4,6-trinitrotoluene)	GSTU24-OE	Gunning et al., 2014
GSTU25	At1g17180		Xenobiotic detoxification (TNT, 2,4,6-trinitrotoluene)	GSTU25-OE	Gunning et al., 2014
GSTU26	At1g17190		Xenobiotic detoxification (herbicides)	None	Nutricati et al., 2006
GSTU27	At3g43800			None	
GSTU28	At1g53680			None	
GSTF2	At4g02520	ATPM24, GST2	Response to bacterial inoculation (*P. syringae*)	None	Lieberherr et al., 2003
			Response to bacterial volatiles (*B. subtilis*)	None	Kwon et al., 2010
GSTF3	At2g02930	GST16		None	
GSTF4	At1g02950	GST31		None	
GSTF5	At1g02940			None	
GSTF6	At1g02930	ERD11, GST1, GSTF3	Response to bacterial inoculation (*P. syringae*)	None	Lieberherr et al., 2003
			Modulation of plant metabolism in response to oxidative stress	*gstf6 gstf7 gstf9 gstf10* RNAi lines	Sappl et al., 2009
			Camalexin biosynthesis (conjugation of GSH with IAN)	GSTF6-OE and *gstf6*	Su et al., 2011
GSTF7	At1g02920	GST11, GSTF8	Modulation of plant metabolism in response to oxidative stress	*gstf6 gstf7 gstf9 gstf10* RNAi lines	Sappl et al., 2009
GSTF8	At2g47730	GST6, GSTF5, GSTF6	Part of the lipid stress response	None	Mueller et al., 2008
			Response to fungal (R. solani) and bacterial (P. syringae) pathogens	None	Gleason et al., 2011
			Response to fungal pathogens (*F. oxysporum*)	None	Thatcher et al., 2015
GSTF9	At2g30860	GLUTTR, GSTF7	Xenobiotic detoxification (CDNB, 1-chloro-2,4-dinitrobenzene)	None	Nutricati et al., 2006
			Indole glucosinolate biosynthesis	None	Wentzell et al., 2007
			Modulation of plant metabolism in response to oxidative stress	*gstf6 gstf7 gstf9 gstf10* RNAi lines	Sappl et al., 2009
			Salt stress response	*gstf9*	Horváth et al., 2015
GSTF10	At2g30870		Indole glucosinolate biosynthesis	None	Wentzell et al., 2007
			Drought and salt stress response	GSTF10-OE and *gstf10* RNA lines	Ryu et al., 2009
			Modulation of plant metabolism in response to oxidative stress	*gstf6 gstf7 gstf9 gstf10* RNAi lines	Sappl et al., 2009
GSTF11	At3g03190	GSTF6	Aliphatic glucosinolate biosynthesis	None	Wentzell et al., 2007
GSTF12	At5g17220	TT19	Flavonoid storage (anthocyanins and proanthocyanidins)	*gstfl12*/*tt19*	Kitamura et al., 2004; Sun et al., 2012
			Response to fungal pathogens (*V. dahliae*)	None	Pantelides et al., 2010
GSTF13	At3g62760			None	
GSTF14	At1g49860		Response to virus infection (BSCTV, beet severe curly top virus)	None	Yang et al., 2013
GSTT1	At5g41210	GST10		None	
GSTT2	At5g41240	GST10B	Systemic acquired resistance	*gstt2*	Banday and Nandi, 2018
GSTT3	At5g41220	GST10C		None	
GSTZ1	At2g02390	GST18, GSTZ1, MAAI	Tyrosine catabolism	None	Edwards and Dixon, 2000
GSTZ2	At2g02380			None	
TCHQD	At1g77290			None	


GSTUs were first associated with plant xenobiotic detoxification, in particular the detoxification of herbicides. It was proposed that high GSTU activity, due to a high endogenous level of expression, was at the center of the observed differential sensitivity to herbicides between cereals (e.g., maize, wheat, and rice) and weeds (Cummins et al., 2011). In *A. thaliana*, it was proposed that *AtGSTU26*, whose expression is induced in response to chloroacetanilide herbicide treatments, could participate in the detoxification of these harmful chemicals by catalyzing their glutathionylation (Nutricati et al., 2006). The xenobiotic detoxification activity of GSTU extends to other chemicals. For instance, a recent study focusing on a major worldwide military pollutant, namely the 2,4,6-trinitrotoluene (TNT), highlighted the role played by GSTUs in detoxifying this harmful and highly persistent pollutant by catalyzing its GSH-conjugation (Gunning et al., 2014). In this study, the authors showed that overexpressing *AtGSTU24* and *AtGSTU25*, two genes whose expression is induced by TNT, was sufficient to enhance the ability of *A. thaliana* plants to withstand and detoxify TNT.

In addition to their role in detoxifying xenobiotic compounds, *A. thaliana* GSTUs were associated with the response to environmental cues. One of the best examples is the response to light signals. This was first demonstrated with *GSTU20*, a gene whose expression is induced under far-red irradiation and inhibited by *phytochrome A* (*phyA*) mutation (Chen et al., 2007). The characterization of gain- and loss-of-function mutant lines suggested the key role that GSTU20 plays in regulating cell elongation and flowering time in response to light (Chen et al., 2007). Similarly, *GSTU17*, whose expression is regulated by different photoreceptors (especially phyA), participates in the modulation of several aspects of seedling development (e.g., hypocotyl elongation, root development, anthocyanin accumulation) (Jiang et al., 2010). Two other GSTUs were also associated with the *A. thaliana* response to light stress, namely *GSTU5* and *GSTU14* (Liu and Li, 2002; Lv et al., 2015).

GSTU17 also plays a role in the response to drought and salt stresses (Chen et al., 2012). The *gstu17* mutation confers a higher tolerance to drought and salt stresses when compared to wild-type plants that could be attributed to an increased accumulation of GSH and ABA within the plant tissues. In this process, GSTU17 acts as a negative component of the stress-mediated signal transduction pathways. Conversely, the overexpression of GSTU19 in *A. thaliana* plants confers tolerance to drought and salt stresses (Xu et al., 2016). The fact that the overexpression of *GSTU19* also confers an increased tolerance to methyl viologen (a pro-oxidant compound) together with increased activity of antioxidant enzymes indicates that GSTU19 may be involved in counteracting the oxidative damages associated with drought or salt stresses. Additionally, the *A. thaliana* response to drought and salt stresses, mediated by the AtRGGA RNA-binding protein, involves *GSTU9* (Ambrosone et al., 2015).

GSTUs are also involved in plant response to biotic stresses. A recent example is the observation that *gstu13* mutants display an enhanced susceptibility, when compared to wild-type plants, toward the fungal pathogens *Erysiphe pisi, Colletotrichum gloeosporioides*, and *Plectosphaerella cucumerina* (Piślewska-Bednarek et al., 2018). It was demonstrated that this phenotype is essentially due to a decrease in the biosynthesis of indole glucosinolates (IG; defense-related compounds found in Brassicaceae), where GSTU13 plays a catalytic role in conjugating GSH to IG precursors, affecting the innate immune system of *A. thaliana* plants. *GSTU16* is another member that was proposed to be part of the plant response to fungal pathogens (e.g., *Verticillium dahliae*) (Pantelides et al., 2010). However, the mechanism by which GSTU16 participates in this response still needs to be addressed.

Both biotic and abiotic stresses lead to the formation of non-enzymatically formed oxylipins, such as phytoprostanes, resulting from the oxidation of several types of lipids (most probably in response to the accumulation of stress-mediated free radicals and reactive oxygen species). These compounds serve as signaling molecules to adapt the plant response to environmental constraints, but may also enhance oxidative stress damages. Thus, their homeostasis should be tightly regulated. Interestingly, it has been shown that GSH-conjugation participates in this process, in particular in response to a *Pseudomonas syringae* infection (Mueller et al., 2008). In addition, the expression of several *GSTU* genes was shown to be responsive to phytoprostanes (i.e., *GSTU7, GSTU18*, and *GSTU24*) indicating that they may regulate phytoprostane homeostasis or be involved in the general detoxification pathways (Mueller et al., 2008; Stotz et al., 2013).

GSTFs constitute the second largest class of Ser-GSTs in plants, with 13 members in *A. thaliana* (Wagner et al., 2002). The GSTF1 sequence that was initially described (Bartling et al., 1993) is no longer present in the final reference genome of this plant.

As GSTUs, GSTFs are associated with the plant response to various abiotic and biotic stresses. For instance, *A. thaliana gstf9* and *gstf10* mutants are more sensitive to a salt stress than wild-type plants (Ryu et al., 2009; Horváth et al., 2015). In contrast, overexpression of *GSTF10* confers higher tolerance to salt. Together these data indicate that the *A. thaliana* GSTF9 and GSTF10 play a positive role in the plant response to a salt stress. However, several reports indicate that the role of *GSTF9* and *GSTF10* extends beyond. *GSTF10* was proposed to play a role in modulating developmental processes, such as the brassinosteroid-independent spontaneous cell death, a mechanism that is mediated by the production of reactive oxygen species (Ryu et al., 2009). *GSTF9* and *GSTF10*, as well as *GSTF11*, are also involved in the biosynthesis of glucosinolates (Wentzell et al., 2007). Whether GSTF9, GSTF10 and GSTF11 act in concert with GSTU13 in this process (Piślewska-Bednarek et al., 2018) remains to be investigated. GSTF9 and GSTF10, together with GSTF6 and GSTF7, also play a role in limiting the metabolic changes that arise during oxidative stress (Sappl et al., 2009).

Additionally, GSTF6 activity is required for the biosynthesis of camalexin, the main phytoalexin (i.e., secondary metabolite with antimicrobial activity) present in *A. thaliana* (Su et al., 2011). The proposed role of GSTF6 in this process is to catalyze the conjugation of GSH on indole-3-acetonitrile (IAN), leading to the formation of GSH-IAN, the main precursor of camalexin biosynthesis. The above-mentioned functional roles of GSTF6 are in agreement with its first proposed biological function, which was to participate in defense mechanisms against pathogens (e.g., *P. syringae*), together with GSTF2 (Lieberherr et al., 2003; Kwon et al., 2010). GSTF8, GSTF12, and GSTF14 activities are associated with the *A. thaliana* response to various pathogens, including viruses (Pantelides et al., 2010; Gleason et al., 2011; Yang et al., 2013; Thatcher et al., 2015). GSTF8 and GSTF9 have been identified as potential actors of the lipid stress response and xenobiotic detoxification machinery, respectively (Nutricati et al., 2006; Mueller et al., 2008).

GSTF12 is another member of the phi class whose function has been extensively studies. GSTF12 is also known as TRANSPARENT TESTA 19 (TT19) and plays a key role in the control of anthocyanin and proanthocyanidin vacuolar accumulation in *A. thaliana* vegetative tissues and seed testa, respectively (Kitamura et al., 2004). In this process, TT19 acts as a carrier to convey these cytosolic flavonoids to the tonoplasts (Sun et al., 2012).

No more physiological information is available for other Ser-GST classes, namely GSTT, GSTZ, and TCHQD (3, 2, and 1 members in *A. thaliana*, respectively). *A. thaliana* GSTT2 seems involved in the modulation of systemic acquired resistance by altering the expression of key genes involved in this process through epigenetic modifications (Banday and Nandi, 2018). According to its capacity to catalyze the isomerisation of maleylacetoacetate into fumarylacetoacetate *in vitro*, AtGSTZ1 is likely involved in tyrosine catabolism, as demonstrated in animals (Edwards and Dixon, 2000). However, there is no mutant described so far and there is a second gene in *A. thaliana*, the biochemical properties of the protein having not yet been explored. Moreover, AtGSTZ1 was also able to catalyze the GSH-dependent dehalogenation of dichloroacetic acid to glyoxylic acid, suggesting other possible functions *in planta*. Finally, there is to date no clear function attributed to the sole member of the TCHQD class, either in *A. thaliana* or in another photosynthetic organisms.

## Conclusion

The GST gene family was subject to a huge genetic expansion in terrestrial plants, with an average number of GSTs around 58 but also in some particular fungi (up to 45 isoforms) (Morel et al., 2013) compared to the 6–17 genes found in bacteria, yeast or mammals. This is linked to expansion within one or several classes as GSTU in plants. Genomic organization and phylogenetic analyses indicate that most duplication events responsible for the expansion of the GSTF and GSTU classes are species-specific. Among Ser-GSTs in plants, the GSTFs and GSTUs are highly represented, and have more diversified primary sequences and catalytic signatures compared to GSTs from the theta, zeta and TCHQD classes. This is likely at the origin of their broader range of activities and set of accommodated substrates/ligands.

According to their stress-inducible expression, it is documented that many GSTFs and GSTUs have functions connected to secondary metabolism, as exemplified by their implication in the vacuolar sequestration of anthocyanin, in the biosynthesis of camalexin, and/or in the binding of biosynthesis intermediates (porphyrin derivatives) or cellular by-products (oxylipins). Their implication in xenobiotic detoxification, such as herbicides, has likely little to do with their ancestral functions, that still remain to be delineated in many cases. However, this has led to the development of biotechnological applications in agriculture and environmental sciences [see the review in this research topic by Perperopoulou et al. (2018)]. The GSTs being targeted for the development of transgenic plants are linked to conferring tolerances against biotic and abiotic stresses or expressing engineered xenobiotic metabolizing enzymes for the bioremediation and detoxification of agrochemicals and pollutants.

To reveal the functions of Ser-GSTs, future studies will have to take into account the redundancy that most probably exists within and between the different classes of Ser-GSTs, which has limited the otherwise powerful reverse-genetic strategy. In order to inventory molecules able to bind GSTs, *in vivo* and *in vitro* ligand-fishing approaches have been used successfully in several cases (Dixon et al., 2011b; Dixon and Edwards, 2018). However, determining the nature of the identified molecules and whether, when and how they are conjugated with GSH or just bound to GSTs is a technically challenging and time-consuming task. For instance, out of the 43 structures of Ser-GSTs available to date in the Protein Data Bank, only 6 are solved with bound ligands. Hence, the validation of the physiological relevance of the detected interactions remains a major challenge.

## Author Contributions

ES-G, AH, and NR carried out the *in silico* genome analyses. SL and OK performed the transcriptomic analyses. ClD and MS performed structural data analyses. KR and ChD compiled data presented in Table 4. All authors participated to the writing of the manuscript.

## Conflict of Interest Statement

The authors declare that the research was conducted in the absence of any commercial or financial relationships that could be construed as a potential conflict of interest.

## References

[B1] Ahmad L. Rylott E. L. Bruce N. C. Edwards R. Grogan G. (2017). Structural evidence for *Arabidopsis* glutathione transferase At GSTF2 functioning as a transporter of small organic ligands. *FEBS Open Bio* 7 122–132. 10.1002/2211-5463.12168 28174680 PMC5292665

[B2] Ahsan N. Lee D.-G. Alam I. Kim P. J. Lee J. J. Ahn Y.-O. (2008). Comparative proteomic study of arsenic-induced differentially expressed proteins in rice roots reveals glutathione plays a central role during As stress. *Proteomics* 8 3561–3576. 10.1002/pmic.200701189 18752204

[B3] Alfenito M. R. (1998). Functional complementation of anthocyanin sequestration in the vacuole by widely divergent glutathione S-transferases. *Plant Cell Online* 10 1135–1150. 10.1105/tpc.10.7.1135 9668133 PMC144053

[B4] Ambrosone A. Batelli G. Nurcato R. Aurilia V. Punzo P. Bangarusamy D. K. (2015). The *Arabidopsis* RNA-binding protein AtRGGA regulates tolerance to salt and drought stress. *Plant Physiol.* 168 292–306. 10.1104/pp.114.255802 25783413 PMC4424017

[B5] Axarli I. Dhavala P. Papageorgiou A. C. Labrou N. E. (2009a). Crystallographic and functional characterization of the fluorodifen-inducible glutathione transferase from *Glycine max* reveals an active site topography suited for diphenylether herbicides and a novel L-site. *J. Mol. Biol.* 385 984–1002. 10.1016/j.jmb.2008.10.084 19014949

[B6] Axarli I. Dhavala P. Papageorgiou A. C. Labrou N. E. (2009b). Crystal structure of *Glycine max* glutathione transferase in complex with glutathione: investigation of the mechanism operating by the Tau class glutathione transferases. *Biochem. J.* 422 247–256. 10.1042/BJ20090224 19538182

[B7] Axarli I. Georgiadou C. Dhavala P. Papageorgiou A. C. Labrou N. E. (2010). Investigation of the role of conserved residues Ser13, Asn48 and Pro49 in the catalytic mechanism of the tau class glutathione transferase from *Glycine max*. *Biochim. Biophys. Acta (BBA) – Proteins Proteom.* 1804 662–667. 10.1016/j.bbapap.2009.10.016 19879385

[B8] Axarli I. Muleta A. W. Vlachakis D. Kossida S. Kotzia G. Maltezos A. (2016). Directed evolution of Tau class glutathione transferases reveals a site that regulates catalytic efficiency and masks co-operativity. *Biochem. J.* 473 559–570. 10.1042/BJ20150930 26637269

[B9] Axarli I. A. Rigden D. J. Labrou N. E. (2004). Characterization of the ligandin site of maize glutathione S-transferase I. *Biochem. J.* 382 885–893. 10.1042/BJ20040298 15196053 PMC1133964

[B10] Banday Z. Z. Nandi A. K. (2018). *Arabidopsis thaliana* GLUTATHIONE- S -TRANSFERASE THETA 2 interacts with RSI1/FLD to activate systemic acquired resistance: GSTT2 interacts with FLD and regulates SAR. *Mol. Plant Pathol.* 19 464–475. 10.1111/mpp.12538 28093893 PMC6638090

[B11] Bartling D. Radzio R. Steiner U. Weiler E. W. (1993). A glutathione S-transferase with glutathione-peroxidase activity from *Arabidopsis thaliana*. Molecular cloning and functional characterization. *Eur. J. Biochem.* 216 579–586. 10.1111/j.1432-1033.1993.tb18177.x 8375395

[B12] Bilang J. Macdonald H. King P. J. Sturm A. (1993). A soluble auxin-binding protein from *Hyoscyamus muticus* is a glutathione S-transferase. *Plant Physiol.* 102 29–34. 10.1104/pp.102.1.29 8108497 PMC158743

[B13] Board P. G. Coggan M. Chelvanayagam G. Easteal S. Jermiin L. S. Schulte G. K. (2000). Identification, characterization, and crystal structure of the omega class glutathione transferases. *J. Biol. Chem.* 275 24798–24806. 10.1074/jbc.M001706200 10783391

[B14] Board P. G. Menon D. (2013). Glutathione transferases, regulators of cellular metabolism and physiology. *Biochim. Biophys. Acta (BBA) – Gen. Subjects* 1830 3267–3288. 10.1016/j.bbagen.2012.11.019 23201197

[B15] Booth J. Boyland E. Sims P. (1961). An enzyme from rat liver catalysing conjugations with glutathione. *Biochem. J.* 79 516–524. 10.1042/bj0790516 16748905 PMC1205680

[B16] Bryant D. Cummins I. Dixon D. P. Edwards R. (2006). Cloning and characterization of a theta class glutathione transferase from the potato pathogen *Phytophthora infestans*. *Phytochemistry* 67 1427–1434. 10.1016/j.phytochem.2006.05.012 16797619

[B17] Buetler T. M. Eaton D. L. (1992). Complementary DNA cloning, messenger RNA expression, and induction of alpha-class glutathione S-transferases in mouse tissues. *Cancer Res.* 52 314–318. 1728405

[B18] Burmeister C. Lüersen K. Heinick A. Hussein A. Domagalski M. Walter R. D. (2008). Oxidative stress in *Caenorhabditis elegans*?: protective effects of the Omega class glutathione transferase (GSTO-1). *FASEB J.* 22 343–354. 10.1096/fj.06-7426com 17901115

[B19] Chen C.-Y. Ho S.-S. Kuo T.-Y. Hsieh H.-L. Cheng Y.-S. (2017). Structural basis of jasmonate-amido synthetase FIN219 in complex with glutathione S-transferase FIP1 during the JA signal regulation. *Proc. Natl. Acad. Sci. U.S.A.* 114 E1815–E1824. 10.1073/pnas.1609980114 28223489 PMC5347581

[B20] Chen I.-C. Huang I.-C. Liu M.-J. Wang Z.-G. Chung S.-S. Hsieh H.-L. (2007). Glutathione S-transferase interacting with far-red insensitive 219 is involved in phytochrome A-mediated signaling in *Arabidopsis*. *Plant Physiol.* 143 1189–1202. 10.1104/pp.106.094185 17220357 PMC1820923

[B21] Chen J.-H. Jiang H.-W. Hsieh E.-J. Chen H.-Y. Chien C.-T. Hsieh H.-L. (2012). Drought and salt stress tolerance of an arabidopsis glutathione S-transferase U17 knockout mutant are attributed to the combined effect of glutathione and abscisic acid. *Plant Physiol.* 158 340–351. 10.1104/pp.111.181875 22095046 PMC3252094

[B22] Cho H.-Y. Lee H. J. Kong K.-H. (2007). A phi class glutathione S-transferase from *Oryza sativa* (OsGSTF5): molecular cloning, expression and biochemical characteristics. *J. Biochem. Mol. Biol.* 40 511–516. 10.5483/bmbrep.2007.40.4.511 17669266

[B23] Chronopoulou E. Ataya F. S. Pouliou F. Perperopoulou F. Georgakis N. Nianiou-Obeidat I. (2017a). “Structure, evolution and functional roles of plant glutathione transferases,” in *Glutathione in Plant Growth, Development, and Stress Tolerance*, eds Hossain M. A. Mostofa M. G. Diaz-Vivancos P. Burritt D. J. Fujita M. Tran L.-S. P. (Cham: Springer International Publishing), 195–213. 10.1007/978-3-319-66682-2_9

[B24] Chronopoulou E. Georgakis N. Nianiou-Obeidat I. Madesis P. Perperopoulou F. Pouliou F. (2017b). “Plant glutathione transferases in abiotic stress response and herbicide resistance,” in *Glutathione in Plant Growth, Development, and Stress Tolerance*, eds Hossain M. A. Mostofa M. G. Diaz-Vivancos P. Burritt D. J. Fujita M. Tran L.-S. P. (Cham: Springer International Publishing), 215–233. 10.1007/978-3-319-66682-2_10

[B25] Coggan M. Flanagan J. U. Parker M. W. Vichai V. Pearson W. R. Board P. G. (2002). Identification and characterization of GSTT3, a third murine theta class glutathione transferase. *Biochem. J.* 366 323–332. 10.1042/bj20011878 12038961 PMC1222777

[B26] Coleman J. Blake-Kalff M. Davies E. (1997). Detoxification of xenobiotics by plants: chemical modification and vacuolar compartmentation. *Trends Plant Sci.* 2 144–151. 10.1016/S1360-1385(97)01019-4

[B27] Combes B. Stakelum G. S. (1961). A liver enzyme that conjugates sulfobromophtalein sodium with glutathione. *J. Clin. Investig.* 40 981–988. 10.1172/JCI104337 13694895 PMC290815

[B28] Conn S. Curtin C. Bézier A. Franco C. Zhang W. (2008). Purification, molecular cloning, and characterization of glutathione S-transferases (GSTs) from pigmented *Vitis vinifera* L. cell suspension cultures as putative anthocyanin transport proteins. *J. Exp. Bot.* 59 3621–3634. 10.1093/jxb/ern217 18836188 PMC2561157

[B29] Cummins I. Dixon D. P. Freitag-Pohl S. Skipsey M. Edwards R. (2011). Multiple roles for plant glutathione transferases in xenobiotic detoxification. *Drug Metabol. Rev.* 43 266–280. 10.3109/03602532.2011.552910 21425939

[B30] Cummins I. O’Hagan D. Jablonkai I. Cole D. J. Hehn A. Werck-Reichhart D. (2003). Cloning, characterization and regulation of a family of phi class glutathione transferases from wheat. *Plant Mol. Biol.* 52 591–603. 12956529 10.1023/a:1024858218804

[B31] Deponte M. (2013). Glutathione catalysis and the reaction mechanisms of glutathione-dependent enzymes. *Biochim. Biophys. Acta (BBA) – Gen. Subjects* 1830 3217–3266. 10.1016/j.bbagen.2012.09.018 23036594

[B32] DeRidder B. P. (2002). Induction of glutathione S-transferases in *Arabidopsis* by herbicide safeners. *Plant Physiol.* 130 1497–1505. 10.1104/pp.010066 12428014 PMC166668

[B33] Ding N. Wang A. Zhang X. Wu Y. Wang R. Cui H. (2017). Identification and analysis of glutathione S-transferase gene family in sweet potato reveal divergent GST-mediated networks in aboveground and underground tissues in response to abiotic stresses. *BMC Plant Biol.* 17:225. 10.1186/s12870-017-1179-z 29179697 PMC5704550

[B34] Dixon D. P. Cummins I. Cole D. J. Edwards R. (1998). Glutathione-mediated detoxification systems in plants. *Curr. Opin. Plant Biol.* 1 258–266. 10.1016/S1369-5266(98)80114-3 10066594

[B35] Dixon D. P. Edwards R. (2009). Selective binding of glutathione conjugates of fatty acid derivatives by plant glutathione transferases. *J. Biol. Chem.* 284 21249–21256. 10.1074/jbc.M109.020107 19520850 PMC2755848

[B36] Dixon D. P. Edwards R. (2010a). Glutathione transferases. *Arabidopsis Book* 8:e0131. 10.1199/tab.0131 22303257 PMC3244946

[B37] Dixon D. P. Edwards R. (2010b). Roles for stress-inducible lambda glutathione transferases in flavonoid metabolism in plants as identified by ligand fishing. *J. Biol. Chem.* 285 36322–36329. 10.1074/jbc.M110.164806 20841361 PMC2978560

[B38] Dixon D. P. Edwards R. (2018). Protein-ligand fishing in planta for biologically active natural products using glutathione transferases. *Front. Plant Sci.* 9:1659. 10.3389/fpls.2018.01659 30510558 PMC6253249

[B39] Dixon D. P. Hawkins T. Hussey P. J. Edwards R. (2009). Enzyme activities and subcellular localization of members of the *Arabidopsis* glutathione transferase superfamily. *J. Exp. Bot.* 60 1207–1218. 10.1093/jxb/ern365 19174456 PMC2657551

[B40] Dixon D. P. Lapthorn A. Edwards R. (2002). Plant glutathione transferases. *Genome Biol.* 3:REVIEWS3004. 10.1186/gb-2002-3-3-reviews3004 PMC139027 11897031

[B41] Dixon D. P. Lapthorn A. Madesis P. Mudd E. A. Day A. Edwards R. (2008). Binding and glutathione conjugation of porphyrinogens by plant glutathione transferases. *J. Biol. Chem.* 283 20268–20276. 10.1074/jbc.M802026200 18492666

[B42] Dixon D. P. McEwen A. G. Lapthorn A. J. Edwards R. (2003). Forced evolution of a herbicide detoxifying glutathione transferase. *J. Biol. Chem.* 278 23930–23935. 10.1074/jbc.M303620200 12692133

[B43] Dixon D. P. Sellars J. D. Edwards R. (2011a). The *Arabidopsis* phi class glutathione transferase At GSTF2: binding and regulation by biologically active heterocyclic ligands. *Biochem. J.* 438 63–70. 10.1042/BJ20101884 21631432

[B44] Dixon D. P. Steel P. G. Edwards R. (2011b). Roles for glutathione transferases in antioxidant recycling. *Plant Signal. Behav.* 6 1223–1227. 10.4161/psb.6.8.16253 21778824 PMC3260729

[B45] Droog F. (1997). Plant glutathione S-transferases, a tale of theta and tau. *J. Plant Growth Regul.* 16 95–107. 10.1007/PL00006984

[B46] Droog F. N. J. Hooykaas P. J. J. Van Der Zaal B. J. (1995). 2,4-Dichlorophenoxyacetic acid and related chlorinated compounds inhibit two auxin-regulated type-III tobacco glutathione S-transferases. *Plant Physiol.* 107 1139–1146. 10.1104/pp.107.4.1139 12228421 PMC157246

[B47] Edwards R. Dixon D. P. (2000). The role of glutathione transferases in herbicide metabolism. *Environ. Fate Saf. Manage. Agrochem.* 19 216–222.

[B48] Edwards T. E. Bryan C. M. Leibly D. J. Dieterich S. H. Abendroth J. Sankaran B. (2011). Structures of a putative ζ-class glutathione-S-transferase from the pathogenic fungus *Coccidioides immitis*. *Acta Crystallogr. Sect. F Struct. Biol. Cryst. Commun.* 67 1038–1043. 10.1107/S1744309111009493 21904047 PMC3169399

[B49] Fernandez-Canon J. M. Baetscher M. W. Finegold M. Burlingame T. Gibson K. M. Grompe M. (2002). Maleylacetoacetate isomerase (MAAI/GSTZ)-deficient mice reveal a glutathione-dependent nonenzymatic bypass in tyrosine catabolism. *Mol. Cell. Biol.* 22 4943–4951. 10.1128/MCB.22.13.4943-4951.2002 12052898 PMC133921

[B50] Fernández-Cañón J. M. Peñalva M. A. (1998). Characterization of a fungal maleylacetoacetate isomerase gene and identification of its human homologue. *J. Biol. Chem.* 273 329–337. 10.1074/jbc.273.1.329 9417084

[B51] Frear D. S. Swanson H. R. (1970). Biosynthesis of S-(4-ethylamino-6-isopropylamino- 2-s-triazino) glutathione: partial purification and properties of a glutathione S-transferase from corn. *Phytochemistry* 9 2123–2132. 10.1016/S0031-9422(00)85377-7

[B52] Frova C. (2006). Glutathione transferases in the genomics era: new insights and perspectives. *Biomol. Eng.* 23 149–169. 10.1016/j.bioeng.2006.05.020 16839810

[B53] Gallé Á Csiszár J. Secenji M. Guóth A. Cseuz L. Tari I. (2009). Glutathione transferase activity and expression patterns during grain filling in flag leaves of wheat genotypes differing in drought tolerance: response to water deficit. *J. Plant Physiol.* 166 1878–1891. 10.1016/j.jplph.2009.05.016 19615785

[B54] Gleason C. Huang S. Thatcher L. F. Foley R. C. Anderson C. R. Carroll A. J. (2011). Mitochondrial complex II has a key role in mitochondrial-derived reactive oxygen species influence on plant stress gene regulation and defense. *Proc. Natl. Acad. Sci. U.S.A.* 108 10768–10773. 10.1073/pnas.1016060108 21670306 PMC3127871

[B55] Gomez C. Conejero G. Torregrosa L. Cheynier V. Terrier N. Ageorges A. (2011). In vivo grapevine anthocyanin transport involves vesicle-mediated trafficking and the contribution of anthoMATE transporters and GST: anthocyanin trafficking in grapevine. *Plant J.* 67 960–970. 10.1111/j.1365-313X.2011.04648.x 21605207

[B56] Gong H. Jiao Y. Hu W. Pua E. (2005). Expression of glutathione-S-transferase and its role in plant growth and development in vivo and shoot morphogenesis in vitro. *Plant Mol. Biol.* 57 53–66. 10.1007/s11103-004-4516-1 15821868

[B57] Gonneau M. Mornet R. Laloue M. (1998). A Nicotiana plumbaginifolia protein labeled with an azido cytokinin agonist is a glutathione S-transferase. *Physiol. Plant.* 103 114–124. 10.1034/j.1399-3054.1998.1030114.x

[B58] Gonzalez D. Fraichard S. Grassein P. Delarue P. Senet P. Nicolaï A. (2018). Characterization of a *Drosophila* glutathione transferase involved in isothiocyanate detoxification. *Insect Biochem. Mol. Biol.* 95 33–43. 10.1016/j.ibmb.2018.03.004 29578047

[B59] Goodstein D. M. Shu S. Howson R. Neupane R. Hayes R. D. Fazo J. (2012). Phytozome: a comparative platform for green plant genomics. *Nucleic Acids Res.* 40 D1178–D1186. 10.1093/nar/gkr944 22110026 PMC3245001

[B60] Gronwald J. W. Plaisance K. L. (1998). Isolation and characterization of glutathione S-transferase isozymes from sorghum. *Plant Physiol.* 117 877–892. 10.1104/pp.117.3.877 9662530 PMC34942

[B61] Gunning V. Tzafestas K. Sparrow H. Johnston E. J. Brentnall A. S. Potts J. R. (2014). *Arabidopsis* glutathione transferases U24 and U25 exhibit a range of detoxification activities with the environmental pollutant and explosive, 2,4,6-trinitrotoluene. *Plant Physiol.* 165 854–865. 10.1104/pp.114.237180 24733884 PMC4044842

[B62] Habig W. H. Pabst M. J. Fleischner G. Gatmaitan Z. Arias I. M. Jakoby W. B. (1974). The identity of glutathione S-transferase B with ligandin, a major binding protein of liver. *Proc. Natl. Acad. Sci. U.S.A.* 71 3879–3882. 10.1073/pnas.71.10.3879 4139704 PMC434288

[B63] Han J.-B. Li G.-Q. Wan P.-J. Zhu T.-T. Meng Q.-W. (2016). Identification of glutathione S-transferase genes in Leptinotarsa decemlineata and their expression patterns under stress of three insecticides. *Pesticide Biochem. Physiol.* 133 26–34. 10.1016/j.pestbp.2016.03.008 27742358

[B64] Hayes J. D. McLellan L. I. (1999). Glutathione and glutathione-dependent enzymes represent a co-ordinately regulated defence against oxidative stress. *Free Radic. Res.* 31 273–300. 10.1080/10715769900300851 10517533

[B65] Hayes J. D. Pulford D. J. (1995). The glut athione S-transferase supergene family: regulation of GST and the contribution of the lsoenzymes to cancer chemoprotection and drug resistance part I. *Crit. Rev. Biochem. Mol. Biol.* 30 445–520. 10.3109/10409239509083491 8770536

[B66] He G. Guan C.-N. Chen Q.-X. Gou X.-J. Liu W. Zeng Q.-Y. (2016). Genome-wide analysis of the glutathione S-transferase gene family in capsella rubella: identification, expression, and biochemical functions. *Front. Plant Sci.* 7:1325. 10.3389/fpls.2016.01325 27630652 PMC5005422

[B67] Horváth E. Bela K. Papdi C. Gallé Á Szabados L. Tari I. (2015). The role of *Arabidopsis* glutathione transferase F9 gene under oxidative stress in seedlings. *Acta Biol. Hungarica* 66 406–418. 10.1556/018.66.2015.4.5 26616373

[B68] Huang Y. Xun R. Chen G. Xun L. (2008). Maintenance role of a glutathionyl-hydroquinone lyase (PcpF) in pentachlorophenol degradation by *Sphingobium chlorophenolicum* ATCC 39723. *J. Bacteriol.* 190 7595–7600. 10.1128/JB.00489-08 18820023 PMC2583618

[B69] Hurst R. Bao Y. Jemth P. Mannervik B. Williamson G. (1998). Phospholipid hydroperoxide glutathione peroxidase activity of human glutathione transferases. *Biochem. J.* 332(Pt 1), 97–100. 10.1042/bj3320097 9576856 PMC1219456

[B70] Islam S. Rahman I. A. Islam T. Ghosh A. (2017). Genome-wide identification and expression analysis of glutathione S-transferase gene family in tomato: gaining an insight to their physiological and stress-specific roles. *PLoS One* 12:e0187504. 10.1371/journal.pone.01875004 29095889 PMC5667761

[B71] Jakobsson P. J. Morgenstern R. Mancini J. Ford-Hutchinson A. Persson B. (1999). Common structural features of MAPEG – a widespread superfamily of membrane associated proteins with highly divergent functions in eicosanoid and glutathione metabolism. *Protein Sci.* 8 689–692. 10.1110/ps.8.3.689 10091672 PMC2144274

[B72] Jakoby W. B. (1978). The glutathione S-transferases: a group of multifunctional detoxification proteins. *Adv. Enzymol. Relat. Areas Mol. Biol.* 46 383–414. 10.1002/9780470122914.ch6 345769

[B73] Jha B. Sharma A. Mishra A. (2011). Expression of SbGSTU (tau class glutathione S-transferase) gene isolated from *Salicornia brachiata* in tobacco for salt tolerance. *Mol. Biol. Rep.* 38 4823–4832. 10.1007/s11033-010-0625-x 21136169

[B74] Jiang H.-W. Liu M.-J. Chen I.-C. Huang C.-H. Chao L.-Y. Hsieh H.-L. (2010). A glutathione S-transferase regulated by light and hormones participates in the modulation of *Arabidopsis* seedling development. *Plant Physiol.* 154 1646–1658. 10.1104/pp.110.159152 20935176 PMC2996023

[B75] Kao C. W. Bakshi M. Sherameti I. Dong S. Reichelt M. Oelmüller R. (2016). A Chinese cabbage (Brassica campetris subsp. Chinensis) τ-type glutathione-S-transferase stimulates Arabidopsis development and primes against abiotic and biotic stress. *Plant Mol. Biol.* 92 643–659. 10.1007/s11103-016-0531-2 27796720

[B76] Keppler D. (1999). Export pumps for glutathione S-conjugates. *Free Radic. Biol. Med.* 27 985–991. 10.1016/s0891-5849(99)00171-9 10569630

[B77] Ketley J. N. Habig W. H. Jakoby W. B. (1975). Binding of nonsubstrate ligands to the glutathione S-transferases. *J. Biol. Chem.* 250 8670–8673. 1184584

[B78] Khan N. Hu C. Amjad Khan W. Hou X. (2018). Genome-wide identification, classification, and expression divergence of glutathione-transferase family in *Brassica rapa* under multiple hormone treatments. *BioMed. Res. Int.* 2018 1–19. 10.1155/2018/6023457 29992155 PMC5994329

[B79] Kiefer P. M. Copley S. D. (2002). Characterization of the initial steps in the reductive dehalogenation catalyzed by tetrachlorohydroquinone dehalogenase †. *Biochemistry* 41 1315–1322. 10.1021/bi0117504 11802732

[B80] Kilian J. Whitehead D. Horak J. Wanke D. Weinl S. Batistic O. (2007). The AtGenExpress global stress expression data set: protocols, evaluation and model data analysis of UV-B light, drought and cold stress responses: AtGenExpress global abiotic stress data set. *Plant J.* 50 347–363. 10.1111/j.1365-313X.2007.03052.x 17376166

[B81] Kim J. Suh H. Kim S. Kim K. Ahn C. Yim J. (2006). Identification and characteristics of the structural gene for the *Drosophila* eye colour mutant sepia, encoding PDA synthase, a member of the Omega class glutathione S-transferases. *Biochem. J.* 398 451–460. 10.1042/BJ20060424 16712527 PMC1559464

[B82] Kitamura S. Akita Y. Ishizaka H. Narumi I. Tanaka A. (2012). Molecular characterization of an anthocyanin-related glutathione S-transferase gene in cyclamen. *J. Plant Physiol.* 169 636–642. 10.1016/j.jplph.2011.12.011 22251797

[B83] Kitamura S. Shikazono N. Tanaka A. (2004). TRANSPARENT TESTA 19 is involved in the accumulation of both anthocyanins and proanthocyanidins in *Arabidopsis*. *Plant J.* 37 104–114. 10.1046/j.1365-313x.2003.01943.x 14675436

[B84] Kraus P. (1980). Resolution, purification and some properties of three glutathione transferases from rat liver mitochondria. *Hoppe-Seyler’s Z. Physiol. Chem.* 361 9–15. 7358335 10.1515/bchm2.1980.361.1.9

[B85] Kwon Y. S. Ryu C.-M. Lee S. Park H. B. Han K. S. Lee J. H. (2010). Proteome analysis of *Arabidopsis* seedlings exposed to bacterial volatiles. *Planta* 232 1355–1370. 10.1007/s00425-010-1259-x 20820802

[B86] Lallement P.-A. Brouwer B. Keech O. Hecker A. Rouhier N. (2014). The still mysterious roles of cysteine-containing glutathione transferases in plants. *Front. Pharmacol.* 5:192. 10.3389/fphar.2014.00192 25191271 PMC4138524

[B87] Lallement P.-A. Meux E. Gualberto J. M. Dumarcay S. Favier F. Didierjean C. (2015). Glutathionyl-hydroquinone reductases from poplar are plastidial proteins that deglutathionylate both reduced and oxidized glutathionylated quinones. *FEBS Lett.* 589 37–44. 10.1016/j.febslet.2014.11.021 25455804

[B88] Lamoureux G. L. Shimabukuro R. H. Swanson H. R. Frear D. S. (1970). Metabolism of 2-chloro-4-ethylamino-6-isopropylamino-s-triazine (atrazine) in excised sorghum leaf sections. *J. Agric. Food Chem.* 18 81–86. 10.1021/jf60167a029 5524468

[B89] Lan T. Yang Z.-L. Yang X. Liu Y.-J. Wang X.-R. Zeng Q.-Y. (2009). Extensive functional diversification of the populus glutathione S-transferase supergene family. *Plant Cell* 21 3749–3766. 10.1105/tpc.109.070219 19996377 PMC2814494

[B90] Larsen E. S. Alfenito M. R. Briggs W. R. Walbot V. (2003). A carnation anthocyanin mutant is complemented by the glutathione S-transferases encoded by maize Bz2 and petunia An9. *Plant Cell Rep.* 21 900–904. 10.1007/s00299-002-0545-x 12789508

[B91] Lederer B. Böger P. (2003). Binding and protection of porphyrins by glutathione S-transferases of *Zea mays* L. *Biochim. Biophys. Acta (BBA) – Gen. Subjects* 1621 226–233. 10.1016/S0304-4165(03)00073-4 12726999

[B92] Lederer B. Böger P. (2005). A ligand function of glutathione S-transferase, *Z. Naturforsch. C*. 60 166–171. 10.1515/znc-2005-3-403 15948579

[B93] Levi A. J. Arias I. M. (1969). Two hepatic cytoplasmic protein fractions, Y and Z, and their possible role in the hepatic uptake of bilirubin, sulfobromophthalein, and other anions. *J. Clin. Invest.* 48 2156–2167. 10.1172/JCI106182 4980931 PMC297469

[B94] Lieberherr D. Wagner U. Dubuis P.-H. Métraux J.-P. Mauch F. (2003). The rapid induction of glutathione S-transferases AtGSTF2 and AtGSTF6 by avirulent *Pseudomonas* syringae is the result of combined salicylic acid and ethylene signaling. *Plant Cell Physiol.* 44 750–757. 10.1093/pcp/pcg093 12881503

[B95] Litwack G. Ketterer B. Arias I. M. (1971). Ligandin: a hepatic protein which binds steroids, bilirubin, carcinogens and a number of exogenous organic anions. *Nature* 234 466–467. 10.1038/234466a0 4944188

[B96] Liu X.-F. Li J.-Y. (2002). [Characterization of an ultra-violet inducible gene that encodes glutathione S-transferase in *Arabidopsis thaliana*]. *Yi Chuan Xue Bao* 29 458–460. 12043576

[B97] Liu Y.-J. Han X.-M. Ren L.-L. Yang H.-L. Zeng Q.-Y. (2013). Functional divergence of the glutathione S-transferase supergene family in physcomitrella patens reveals complex patterns of large gene family evolution in land plants. *Plant Physiol.* 161 773–786. 10.1104/pp.112.205815 23188805 PMC3561018

[B98] Lv F. Zhou J. Zeng L. Xing D. (2015). β-cyclocitral upregulates salicylic acid signalling to enhance excess light acclimation in *Arabidopsis*. *J. Exp. Bot.* 66 4719–4732. 10.1093/jxb/erv231 25998906

[B99] Mannervik B. Alin P. Guthenberg C. Jensson H. Tahir M. K. Warholm M. (1985). Identification of three classes of cytosolic glutathione transferase common to several mammalian species: correlation between structural data and enzymatic properties. *Proc. Natl. Acad. Sci. U.S.A.* 82 7202–7206. 10.1073/pnas.82.21.7202 3864155 PMC390817

[B100] Mannervik B. Danielson U. H. (1988). Glutathione transferases–structure and catalytic activity. *CRC Crit. Rev. Biochem.* 23 283–337. 3069329 10.3109/10409238809088226

[B101] Marrs K. A. (1996). The functions and regulation of glutathionE S-transferases in plants. *Ann. Rev. Plant Physiol. Plant Mol. Biol.* 47 127–158. 10.1146/annurev.arplant.47.1.127 15012285

[B102] Marrs K. A. Alfenito M. R. Lloyd A. M. Walbot V. (1995). A glutathione S-transferase involved in vacuolar transfer encoded by the maize gene Bronze-2. *Nature* 375 397–400. 10.1038/375397a0 7760932

[B103] Marsh M. Shoemark D. K. Jacob A. Robinson C. Cahill B. Zhou N.-Y. (2008). Structure of bacterial glutathione-S-transferase maleyl pyruvate isomerase and implications for mechanism of isomerisation. *J. Mol. Biol.* 384 165–177. 10.1016/j.jmb.2008.09.028 18824004

[B104] Martínez-Márquez A. Martínez-Esteso M. J. Vilella-Antón M. T. Sellés-Marchart S. Morante-Carriel J. A. Hurtado E. (2017). A tau class glutathione-S-transferase is involved in trans-resveratrol transport out of grapevine cells. *Front. Plant Sci.* 8:1457. 10.3389/fpls.2017.01457 28878794 PMC5573539

[B105] Masai E. Ichimura A. Sato Y. Miyauchi K. Katayama Y. Fukuda M. (2003). Roles of the enantioselective glutathione S-transferases in cleavage of beta-aryl ether. *J. Bacteriol.* 185 1768–1775. 10.1128/jb.185.6.1768-1775.2003 12618439 PMC150126

[B106] Mashiyama S. T. Malabanan M. M. Akiva E. Bhosle R. Branch M. C. Hillerich B. (2014). Large-scale determination of sequence, structure, and function relationships in cytosolic glutathione transferases across the biosphere. *PLoS Biol.* 12:e1001843. 10.1371/journal.pbio.1001843 24756107 PMC3995644

[B107] Menon D. Board P. G. (2013). A role for glutathione transferase omega 1 (GSTO1-1) in the glutathionylation cycle. *J. Biol. Chem.* 288 25769–25779. 10.1074/jbc.M113.487785 23888047 PMC3764784

[B108] Meux E. Morel M. Lamant T. Gérardin P. Jacquot J.-P. Dumarçay S. (2013). New substrates and activity of Phanerochaete chrysosporium Omega glutathione transferases. *Biochimie* 95 336–346. 10.1016/j.biochi.2012.10.003 23063695

[B109] Meux E. Prosper P. Ngadin A. Didierjean C. Morel M. Dumarçay S. (2011). Glutathione transferases of *Phanerochaete chrysosporium*: S-glutathionyl-p-hydroquinone reductase belongs to a new structural class. *J. Biol. Chem.* 286 9162–9173. 10.1074/jbc.M110.194548 21177852 PMC3059006

[B110] Momose M. Itoh Y. Umemoto N. Nakayama M. Ozeki Y. (2013). Reverted glutathione S-transferase-like genes that influence flower color intensity of carnation (*Dianthus caryophyllus* L.) originated from excision of a transposable element. *Breed. Sci.* 63 435–440. 10.1270/jsbbs.63.435 24399917 PMC3859356

[B111] Monticolo F. Colantuono C. Chiusano M. L. (2017). Shaping the evolutionary tree of green plants: evidence from the GST family. *Sci. Rep.* 7:14363. 10.1038/s41598-017-14316-w 29084977 PMC5662610

[B112] Moons A. (2003). Osgstu3 and osgtu4, encoding tau class glutathione S-transferases, are heavy metal- and hypoxic stress-induced and differentially salt stress-responsive in rice roots 1. *FEBS Lett.* 553 427–432. 10.1016/S0014-5793(03)01077-9 14572664

[B113] Moons A. (2005). “Regulatory and functional interactions of plant growth regulators and plant glutathione s-transferases (GSTs),” in *Vitamins & Hormones*, (Amsterdam: Elsevier), 155–202. 10.1016/S0083-6729(05)72005-7 16492471

[B114] Morel M. Meux E. Mathieu Y. Thuillier A. Chibani K. Harvengt L. (2013). Xenomic networks variability and adaptation traits in wood decaying fungi: fungal xenomic networks. *Microb. Biotechnol.* 6 248–263. 10.1111/1751-7915.12015 23279857 PMC3815920

[B115] Mueller L. A. Goodman C. D. Silady R. A. Walbot V. (2000). AN9, a petunia glutathione S-transferase required for anthocyanin sequestration, is a flavonoid-binding protein. *Plant Physiol.* 123 1561–1570. 10.1104/pp.123.4.1561 10938372 PMC59113

[B116] Mueller S. Hilbert B. Dueckershoff K. Roitsch T. Krischke M. Mueller M. J. (2008). General detoxification and stress responses are mediated by oxidized lipids through TGA transcription factors in *Arabidopsis*. *Plant Cell Online* 20 768–785. 10.1105/tpc.107.054809 18334669 PMC2329937

[B117] Munyampundu J.-P. Xu Y.-P. Cai X.-Z. (2016). Phi class of glutathione S-transferase gene superfamily widely exists in nonplant taxonomic groups. *Evol. Bioinform.* 12:EBO.S35909. 10.4137/EBO.S35909 26884677 PMC4750895

[B118] Navrot N. Collin V. Gualberto J. Gelhaye E. Hirasawa M. Rey P. (2006). Plant glutathione peroxidases are functional peroxiredoxins distributed in several subcellular compartments and regulated during biotic and abiotic stresses. *Plant Physiol.* 142 1364–1379. 10.1104/pp.106.089458 17071643 PMC1676047

[B119] Neuefeind T. Huber R. Dasenbrock H. Prade L. Bieseler B. (1997a). Crystal structure of herbicide-detoxifying maize glutathione S-transferase-I in complex with lactoylglutathione: evidence for an induced-fit mechanism. *J. Mol. Biol.* 274 446–453. 10.1006/jmbi.1997.1402 9417926

[B120] Neuefeind T. Huber R. Reinemer P. Knäblein J. Prade L. Mann K. (1997b). Cloning, sequencing, crystallization and X-ray structure of glutathione S-transferase-III from *Zea mays* var. mutin: a leading enzyme in detoxification of maize herbicides. *J. Mol. Biol.* 274 577–587. 10.1006/jmbi.1997.1401 9417936

[B121] Nutricati E. Miceli A. Blando F. De Bellis L. (2006). Characterization of two *Arabidopsis* thaliana glutathione S-transferases. *Plant Cell Rep.* 25 997–1005. 10.1007/s00299-006-0146-1 16538523

[B122] Pantelides I. S. Tjamos S. E. Paplomatas E. J. (2010). Ethylene perception via ETR1 is required in *Arabidopsis* infection by *Verticillium dahliae*. *Mol. Plant Pathol.* 11 191–202. 10.1111/j.1364-3703.2009.00592.x 20447269 PMC6640466

[B123] Pégeot H. Koh C. S. Petre B. Mathiot S. Duplessis S. Hecker A. (2014). The poplar Phi class glutathione transferase: expression, activity and structure of GSTF1. *Front. Plant Sci.* 5:712. 10.3389/fpls.2014.00712 25566286 PMC4274894

[B124] Pégeot H. Mathiot S. Perrot T. Gense F. Hecker A. Didierjean C. (2017). Structural plasticity among glutathione transferase Phi members: natural combination of catalytic residues confers dual biochemical activities. *FEBS J.* 284 2442–2463. 10.1111/febs.14138 28622459

[B125] Pemble S. E. Taylor J. B. (1992). An evolutionary perspective on glutathione transferases inferred from class-theta glutathione transferase cDNA sequences. *Biochem. J.* 287(Pt 3), 957–963. 10.1042/bj2870957 1445253 PMC1133100

[B126] Perperopoulou F. Pouliou F. Labrou N. E. (2018). Recent advances in protein engineering and biotechnological applications of glutathione transferases. *Crit. Rev. Biotechnol.* 38 511–528. 10.1080/07388551.2017.1375890 28936894

[B127] Pettersen E. F. Goddard T. D. Huang C. C. Couch G. S. Greenblatt D. M. Meng E. C. (2004). UCSF chimera–a visualization system for exploratory research and analysis. *J. Comput. Chem.* 25 1605–1612. 10.1002/jcc.20084 15264254

[B128] Piślewska-Bednarek M. Nakano R. T. Hiruma K. Pastorczyk M. Sanchez-Vallet A. Singkaravanit-Ogawa S. (2018). Glutathione transferase U13 functions in pathogen-triggered glucosinolate metabolism. *Plant Physiol.* 176 538–551. 10.1104/pp.17.01455 29122987 PMC5761798

[B129] Plomion C. Aury J.-M. Amselem J. Leroy T. Murat F. Duplessis S. (2018). Oak genome reveals facets of long lifespan. *Nat. Plants* 4 440–452. 10.1038/s41477-018-0172-3 29915331 PMC6086335

[B130] Prade L. Huber R. Bieseler B. (1998). Structures of herbicides in complex with their detoxifying enzyme glutathione S-transferase – explanations for the selectivity of the enzyme in plants. *Structure* 6 1445–1452. 10.1016/S0969-2126(98)00143-9 9817846

[B131] Rahantaniaina M.-S. Li S. Chatel-Innocenti G. Tuzet A. Mhamdi A. Vanacker H. (2017). Glutathione oxidation in response to intracellular H_2_O_2_: key but overlapping roles for dehydroascorbate reductases. *Plant Signal Behav.* 12:e1356531. 10.1080/15592324.2017.1356531 28782990 PMC5616140

[B132] Reddy G. V. B. Gold M. H. (2001). Purification and characterization of glutathione conjugate reductase: a component of the tetrachlorohydroquinone reductive dehalogenase system from phanerochaete chrysosporium. *Arch. Biochem. Biophys.* 391 271–277. 10.1006/abbi.2001.2417 11437359

[B133] Reid R. Gridley K. Kawamata Y. Zhu Y. (2013). Arsenite elicits anomalous sulfur starvation responses in barley. *Plant Physiol.* 162 401–409. 10.1104/pp.113.216937 23482871 PMC3641219

[B134] Reinemer P. Prade L. Hof P. Neuefeind T. Huber R. Zettl R. (1996). Three-dimensional structure of glutathione S-transferase from *Arabidopsis thaliana* at 2.2 Å resolution: structural characterization of herbicide-conjugating plant glutathione S-transferases and a novel active site architecture. *J. Mol. Biol.* 255 289–309. 10.1006/jmbi.1996.0024 8551521

[B135] Rinaldi C. Kohler A. Frey P. Duchaussoy F. Ningre N. Couloux A. (2007). Transcript profiling of poplar leaves upon infection with compatible and incompatible strains of the foliar rust *Melampsora larici*-populina. *Plant Physiol.* 144 347–366. 10.1104/pp.106.094987 17400708 PMC1913798

[B136] Rouhier N. Jacquot J.-P. (2005). The plant multigenic family of thiol peroxidases. *Free Radic. Biol. Med.* 38 1413–1421. 10.1016/j.freeradbiomed.2004.07.037 15890615

[B137] Rouhier N. Lemaire S. D. Jacquot J.-P. (2008). The role of glutathione in photosynthetic organisms: emerging functions for glutaredoxins and glutathionylation. *Ann. Rev. Plant Biol.* 59 143–166. 10.1146/annurev.arplant.59.032607.092811 18444899

[B138] Ryu H. Y. Kim S. Y. Park H. M. You J. Y. Kim B. H. Lee J. S. (2009). Modulations of AtGSTF10 expression induce stress tolerance and BAK1-mediated cell death. *Biochem. Biophys. Res. Commun.* 379 417–422. 10.1016/j.bbrc.2008.11.156 19118534

[B139] Sappl P. G. Carroll A. J. Clifton R. Lister R. Whelan J. Harvey Millar A. (2009). The *Arabidopsis* glutathione transferase gene family displays complex stress regulation and co-silencing multiple genes results in altered metabolic sensitivity to oxidative stress: genomic and reverse genetic analysis of plant GSTs. *Plant J.* 58 53–68. 10.1111/j.1365-313X.2008.03761.x 19067976

[B140] Sappl P. G. Oñate-Sánchez L. Singh K. B. Millar A. H. (2004). Proteomic analysis of glutathione S -transferases of *Arabidopsis thaliana* reveals differential salicylic acid-induced expression of the plant-specific phi and tau classes. *Plant Mol. Biol.* 54 205–219. 10.1023/B:PLAN.0000028786.57439.b3 15159623

[B141] Schmid M. Davison T. S. Henz S. R. Pape U. J. Demar M. Vingron M. (2005). A gene expression map of *Arabidopsis thaliana* development. *Nat. Genet.* 37 501–506. 10.1038/ng1543 15806101

[B142] Schwartz M. Didierjean C. Hecker A. Girardet J.-M. Morel-Rouhier M. Gelhaye E. (2016). Crystal structure of *Saccharomyces cerevisiae* ECM4, a Xi-class glutathione transferase that reacts with glutathionyl-(hydro)quinones. *PLoS One* 11:e0164678. 10.1371/journal.pone.0164678 27736955 PMC5063366

[B143] Shah D. M. Hironaka C. M. Wiegand R. C. Harding E. I. Krivi G. G. Tiemeier D. C. (1986). Structural analysis of a maize gene coding for glutathione-S-transferase involved in herbicide detoxification. *Plant Mol. Biol.* 6 203–211. 10.1007/BF00015226 24307319

[B144] Shao Y. Lv Z. Li C. Zhang W. Duan X. Qiu Q. (2017). Molecular cloning and functional characterization of theta class glutathione S-transferase from *Apostichopus japonicus*. *Fish Shellf. Immunol.* 63 31–39. 10.1016/j.fsi.2017.02.004 28185912

[B145] Skopelitou K. Muleta A. W. Papageorgiou A. C. Chronopoulou E. Labrou N. E. (2015). Catalytic features and crystal structure of a tau class glutathione transferase from *Glycine max* specifically upregulated in response to soybean mosaic virus infections. *Biochim. Biophys. Acta (BBA) – Proteins Proteom.* 1854 166–177. 10.1016/j.bbapap.2014.11.008 25479053

[B146] Skopelitou K. Muleta A. W. Pavli O. Skaracis G. N. Flemetakis E. Papageorgiou A. C. (2012). Overlapping protective roles for glutathione transferase gene family members in chemical and oxidative stress response in *Agrobacterium tumefaciens*. *Funct. Integr. Genomics* 12 157–172. 10.1007/s10142-011-0248-x 21909786

[B147] Smith A. P. DeRidder B. P. Guo W.-J. Seeley E. H. Regnier F. E. Goldsbrough P. B. (2004). Proteomic analysis of *Arabidopsis* glutathione S-transferases from benoxacor- and copper-treated seedlings. *J. Biol. Chem.* 279 26098–26104. 10.1074/jbc.M402807200 15069083

[B148] Smith A. P. Nourizadeh S. D. Peer W. A. Xu J. Bandyopadhyay A. Murphy A. S. (2003). *Arabidopsis* AtGSTF2 is regulated by ethylene and auxin, and encodes a glutathione S-transferase that interacts with flavonoids. *Plant J.* 36 433–442. 10.1046/j.1365-313x.2003.01890.x 14617075

[B149] Soranzo N. Sari Gorla M. Mizzi L. De Toma G. Frova C. (2004). Organisation and structural evolution of the rice glutathione S-transferase gene family. *Mol. Genet. Genomics* 271 511–521. 10.1007/s00438-004-1006-8 15069639

[B150] Srivastava D. Verma G. Chauhan A. S. Pande V. Chakrabarty D. (2019). Rice (*Oryza sativa* L.) tau class glutathione S -transferase (OsGSTU30) overexpression in *Arabidopsis thaliana* modulates a regulatory network leading to heavy metal and drought stress tolerance. *Metallomics* 11 375–389. 10.1039/c8mt00204e 30516767

[B151] Stotz H. U. Mueller S. Zoeller M. Mueller M. J. Berger S. (2013). TGA transcription factors and jasmonate-independent COI1 signalling regulate specific plant responses to reactive oxylipins. *J. Exp. Bot.* 64 963–975. 10.1093/jxb/ers389 23349138 PMC3580818

[B152] Su T. Xu J. Li Y. Lei L. Zhao L. Yang H. (2011). Glutathione-indole-3-acetonitrile is required for camalexin biosynthesis in *Arabidopsis thaliana*. *Plant Cell* 23 364–380. 10.1105/tpc.110.079145 21239642 PMC3051237

[B153] Sun Y. Li H. Huang J.-R. (2012). Arabidopsis TT19 functions as a carrier to transport anthocyanin from the cytosol to tonoplasts. *Mol. Plant* 5 387–400. 10.1093/mp/ssr110 22201047

[B154] Tang A. H. Tu C. P. (1994). Biochemical characterization of *Drosophila* glutathione S-transferases D1 and D21. *J. Biol. Chem.* 269 27876–27884. 7961718

[B155] Thatcher L. F. Kamphuis L. G. Hane J. K. Oñate-Sánchez L. Singh K. B. (2015). The *Arabidopsis* KH-domain RNA-binding protein ESR1 functions in components of jasmonate signalling, unlinking growth restraint and resistance to stress. *PLoS One* 10:e0126978. 10.1371/journal.pone.0126978 25985302 PMC4436139

[B156] Thom R. Cummins I. Dixon D. P. Edwards R. Cole D. J. Lapthorn A. J. (2002). Structure of a tau class glutathione S-transferase from wheat active in herbicide detoxification. *Biochemistry* 41 7008–7020. 10.1021/bi015964x 12033934

[B157] Thom R. Dixon D. P. Edwards R. Cole D. J. Lapthorn A. J. (2001). The structure of a zeta class glutathione S-transferase from *Arabidopsis thaliana*: characterisation of a GST with novel active-site architecture and a putative role in tyrosine catabolism. *J. Mol. Biol.* 308 949–962. 10.1006/jmbi.2001.4638 11352584

[B158] Tiwari V. Patel M. K. Chaturvedi A. K. Mishra A. Jha B. (2016). Functional characterization of the tau class glutathione-S-transferases gene (SbGSTU) promoter of salicornia brachiata under salinity and osmotic stress. *PLoS One* 11:e0148494. 10.1371/journal.pone.0148494 26885663 PMC4757536

[B159] Tossounian M.-A. Van Molle I. Wahni K. Jacques S. Gevaert K. Van Breusegem F. (2018). Disulfide bond formation protects *Arabidopsis thaliana* glutathione transferase tau 23 from oxidative damage. *Biochim. Biophys. Acta (BBA) – Gen. Subjects* 1862 775–789. 10.1016/j.bbagen.2017.10.007 29031766

[B160] Tripathi A. Indoliya Y. Tiwari M. Tiwari P. Srivastava D. Kumar Verma P. (2014). Transformed yeast (*Schizosaccharomyces pombe*) overexpressing rice Tau class glutathione S-transferase (OsGSTU30 and OsGSTU41) shows enhanced resistance to hexavalent chromium. *Metallomics* 6 1549–1557. 10.1039/C4MT00083H 24968244

[B161] Valenzuela-Chavira I. Contreras-Vergara C. A. Arvizu-Flores A. A. Serrano-Posada H. Lopez-Zavala A. A. García-Orozco K. D. (2017). Insights into ligand binding to a glutathione S-transferase from mango: structure, thermodynamics and kinetics. *Biochimie* 135 35–45. 10.1016/j.biochi.2017.01.005 28104507 PMC5346462

[B162] Wagner U. Edwards R. Dixon D. P. Mauch F. (2002). Probing the diversity of the *Arabidopsis* glutathione S-transferase gene family. *Plant Mol. Biol.* 49 515–532. 12090627 10.1023/a:1015557300450

[B163] Wentzell A. M. Rowe H. C. Hansen B. G. Ticconi C. Halkier B. A. Kliebenstein D. J. (2007). Linking metabolic QTLs with network and cis-eQTLs controlling biosynthetic pathways. *PLoS Genet.* 3:e162. 10.1371/journal.pgen.0030162 17941713 PMC1976331

[B164] Wiegand R. C. Shah D. M. Mozer T. J. Harding E. I. Diaz-Collier J. Saunders C. (1986). Messenger RNA encoding a glutathione-S-transferase responsible for herbicide tolerance in maize is induced in response to safener treatment. *Plant Mol. Biol.* 7 235–243. 10.1007/BF00752897 24302366

[B165] Willett S. W. Copley S. D. (1996). Identification and localization of a stable sulfenic acid in peroxide-treated tetrachlorohydroquinone dehalogenase using electrospray mass spectrometry. *Chem. Biol.* 3 851–857. 10.1016/S1074-5521(96)90071-X 8939704

[B166] Xu J. Tian Y.-S. Xing X.-J. Peng R.-H. Zhu B. Gao J.-J. (2016). Over-expression of AtGSTU19 provides tolerance to salt, drought and methyl viologen stresses in *Arabidopsis*. *Physiol. Plant.* 156 164–175. 10.1111/ppl.12347 25975461

[B167] Xu J. Zheng A.-Q. Xing X.-J. Chen L. Fu X.-Y. Peng R.-H. (2018). Transgenic *Arabidopsis* plants expressing grape glutathione S-Transferase gene (VvGSTF13) show enhanced tolerance to abiotic stress. *Biochemistry* 83 755–765. 10.1134/S0006297918060135 30195332

[B168] Xun L. Belchik S. M. Xun R. Huang Y. Zhou H. Sanchez E. (2010). S-Glutathionyl-(chloro)hydroquinone reductases: a novel class of glutathione transferases. *Biochem. J.* 428 419–427. 10.1042/BJ20091863 20388120 PMC2997670

[B169] Xun L. Topp E. Orser C. S. (1992). Purification and characterization of a tetrachloro-p-hydroquinone reductive dehalogenase from a *Flavobacterium* sp. *J. Bacteriol.* 174 8003–8007. 10.1128/jb.174.24.8003-8007.1992 1459949 PMC207537

[B170] Yamamoto K. Nagaoka S. Banno Y. Aso Y. (2009). Biochemical properties of an omega-class glutathione S-transferase of the silkmoth, *Bombyx mori*. *Compar. Biochem. Physiol. Part C Toxicol. Pharmacol.* 149 461–467. 10.1016/j.cbpc.2008.10.108 19022397

[B171] Yamazaki M. Shibata M. Nishiyama Y. Springob K. Kitayama M. Shimada N. (2008). Differential gene expression profiles of red and green forms of *Perilla frutescens* leading to comprehensive identification of anthocyanin biosynthetic genes: anthocyanin biosynthetic genes from *Perilla*. *FEBS J.* 275 3494–3502. 10.1111/j.1742-4658.2008.06496.x 18513325

[B172] Yang G. Xu Z. Peng S. Sun Y. Jia C. Zhai M. (2016). In planta characterization of a tau class glutathione S-transferase gene from Juglans regia (JrGSTTau1) involved in chilling tolerance. *Plant Cell Rep.* 35 681–692. 10.1007/s00299-015-1912-8 26687965

[B173] Yang L.-P. Fang Y.-Y. An C.-P. Dong L. Zhang Z.-H. Chen H. (2013). C2-mediated decrease in DNA methylation, accumulation of siRNAs, and increase in expression for genes involved in defense pathways in plants infected with beet severe curly top virus. *Plant J.* 73 910–917. 10.1111/tpj.12081 23176533

[B174] Yang Q. Han X.-M. Gu J.-K. Liu Y.-J. Yang M.-J. Zeng Q.-Y. (2019). Functional and structural profiles of GST gene family from three *Populus* species reveal the sequence-function decoupling of orthologous genes. *New Phytol.* 221 1060–1073. 10.1111/nph.15430 30204242

[B175] Zakharyan R. A. Sampayo-Reyes A. Healy S. M. Tsaprailis G. Board P. G. Liebler D. C. (2001). Human monomethylarsonic acid (MMA(V)) reductase is a member of the glutathione-S-transferase superfamily. *Chem. Res. Toxicol.* 14 1051–1057. 10.1021/tx010052h 11511179

[B176] Zettl R. Schell J. Palme K. (1994). Photoaffinity labeling of *Arabidopsis thaliana* plasma membrane vesicles by 5-azido-[7-3H]indole-3-acetic acid: identification of a glutathione S-transferase. *Proc. Natl. Acad. Sci. U.S.A.* 91 689–693. 10.1073/pnas.91.2.689 8290582 PMC43014

[B177] Zhao J. (2015). Flavonoid transport mechanisms: how to go, and with whom. *Trends Plant Sci.* 20 576–585. 10.1016/j.tplants.2015.06.007 26205169

